# Chromofungin Ameliorates the Progression of Colitis by Regulating Alternatively Activated Macrophages

**DOI:** 10.3389/fimmu.2017.01131

**Published:** 2017-09-15

**Authors:** Nour Eissa, Hayam Hussein, Laëtitia Kermarrec, Jasmine Grover, Marie-Hélène Et Metz-Boutigue, Charles N. Bernstein, Jean-Eric Ghia

**Affiliations:** ^1^Immunology Department, University of Manitoba, Winnipeg, MB, Canada; ^2^Children’s Hospital Research Institute of Manitoba, University of Manitoba, Winnipeg, MB, Canada; ^3^Department of Veterinary Clinical Sciences, College of Veterinary Medicine, Ohio State University, Columbus, OH, United States; ^4^INSERM U977, Biomatériaux et Ingéniérie tissulaire, Institut Leriche 2éme étage, Hôpital Civil, Porte de l’Hôpital, Strasbourg, France; ^5^Rady Faculty of Health Sciences, Department of Internal Medicine, Section of Gastroenterology, University of Manitoba, Winnipeg, MB, Canada; ^6^University of Manitoba IBD Clinical and Research Centre, Winnipeg, MB, Canada

**Keywords:** chromogranin-A, gut-derived peptides, oxidative stress, mucosal drug action, epithelial homeostasis, macrophages switch, anti-inflammatory molecules, epithelial barrier

## Abstract

Ulcerative colitis (UC) is characterized by a functional dysregulation of alternatively activated macrophage (AAM) and intestinal epithelial cells (IECs) homeostasis. Chromogranin-A (CHGA) secreted by neuroendocrine cells is implicated in intestinal inflammation and immune dysregulation. CHGA undergoes proteolytic processing to generate CHGA-derived peptides. Chromofungin (CHR: CHGA_47–66_) is a short CHGA-derived peptide encoded by CHGA Exon-IV and is involved in innate immune regulation, but the basis is poorly investigated. We investigated the expression of CHR in colonic tissue of patients with active UC and assessed the effects of the CHR in dextran sulfate sodium (DSS) colitis in mice and on macrophages and human colonic epithelial cells. We found that mRNA expression of CHR correlated positively with mRNA levels of AAM markers and gene expression of tight junction (TJ) proteins and negatively with mRNA levels of interleukin (*IL*)*-8, IL-18*, and collagen in patients with active UC. Moreover, AAM markers correlated positively with gene expression of TJ proteins and negatively with *IL-8, IL-18*, and collagen gene expression. Experimentally, intracolonic administration of CHR protected against DSS-induced colitis by priming macrophages into AAM, reducing colonic collagen deposition, and maintaining IECs homeostasis. This effect was associated with a significant increase of AAM markers, reduction of colonic IL-18 release and conservation of gene expression of TJ proteins. *In vitro*, CHR enhanced AAM polarization and increased the production of anti-inflammatory mediators. CHR-treated AAM conditioned medium increased Caco-2 cell migration, viability, proliferation, and mRNA levels of TJ proteins, and decreased oxidative stress-induced apoptosis and proinflammatory cytokines release. Direct CHR treatments had the same effect. In conclusion, CHR treatment reduces the severity of colitis and the inflammatory process via enhancing AAM functions and maintaining IECs homeostasis. CHR is involved in the pathogenesis of inflammation in experimental colitis. These findings provide insight into the mechanisms of colonic inflammation and could lead to new therapeutic strategies for UC.

## Introduction

Crohn’s disease and ulcerative colitis (UC) are the two main forms of inflammatory bowel disease (IBD) in humans (1). The etiology of IBD is unknown, but evidence suggests that the abnormal immune response within the intestinal wall is directed against luminal bacterial antigens inducing intestinal tissue damage (2). Analysis of proinflammatory and anti-inflammatory pathways in IBD patients have demonstrated dysregulation in the immune responses associated with an altered balance between inflammatory, regulatory and anti-inflammatory cytokines (3). Macrophages implicated in presenting antigens to T and B cells are important cells regulating the host innate and adaptive immune responses (4). In IBD, macrophages play a crucial role in the resolution of tissue injury and promotion of tissue repair (5, 6). Two main categories of macrophages are described, the classical-activated macrophages (CAMs) which generate Th1-related cytokines (interferon-γ, tumor necrosis factor-α) response, and the alternatively activated macrophages (AAMs) linked to a Th2-related cytokines [interleukin (IL)-4 and IL-13] response (7). AAMs produce anti-inflammatory molecules (IL-10, arginase) and play a major role in the suppression of inflammation and tissue remodeling/repair (8). AAMs have been reported to attenuate experimental inflammation in the gut (9–11).

Intestinal epithelial cells (IECs) form a crucial first line of physical defense between the mucosa and the luminal milieu. Tight junctions (TJ) proteins are mainly responsible for the epithelial barrier function that includes selective transport of water, ions, and nutrients by forming an uninterrupted intercellular barrier between the epithelial cells (12). Thus, defects in intestinal epithelial TJ barrier are important contributing factors for the development of intestinal inflammation and lead to an amplified inflammatory response due to an increased passage of antigens into the colonic mucosa (13, 14). Furthermore, in persons with IBD, IECs secrete a significant quantity of chemokines (i.e., IL-8) which cause excessive recruitment and transmigration of innate immune cells and proinflammatory cytokines, including IL-18 (15, 16). Additionally, high collagen production by IECs, colonocytes, and fibroblasts favors intestinal fibrosis associated with stricture formation, which is a significant complication seen in persons with IBD (16). Furthermore, oxidative stress, and subsequent epithelial apoptosis, is a fundamental feature of colitis (17).

Chromogranin-A (CHGA), a member of the granin family of proteins (18), is an acidic protein distributed ubiquitously in vesicles of secretory cells of the enteric, endocrine, and immune systems (18). CHGA is the precursor of biologically active peptides implicated in several biological functions (19) by regulating the endocrine, the cardiovascular, and the immune systems (18, 20). The protein is cleaved at multiple dibasic sites and exposed to an extensive degree of intracellular and extracellular proteolytic processing, particularly at the N- and C-terminal regions (21) to generate CHGA-derived peptides including chromofungin (CHR: CHGA_47–66_). CHR is an active short peptide encoded by the CHGA exon-IV (18) that possesses antimicrobial activity (22–24) and immune regulatory functions (25). Although CHR has antimicrobial activity, it is a non-hemolytic peptide, suggesting its non-toxicity (26). Moreover, CHGA_47–57_ peptide, which is a part of CHR, contains a cell adhesion site for fibroblasts and smooth muscle cells (26). Furthermore, CHR displays pronociceptive and antinociceptive effects in a model of somatovisceral pain through various mechanisms involving the corticotropin-releasing factor pathway, action on sensory neurotransmitter and direct or indirect regulation of inflammatory cells (27). Recently, CHR has been described as a post-conditioning agent against ischemia/reperfusion (I/R) damages through the activation of prosurvival kinases and an increased miRNA-21 expression (28).

Although CHGA and its derived peptides are implicated in various inflammatory diseases including gut inflammation (29–33), there are no available data demonstrating the effects of CHR on AAM and IECs homeostasis during the progression of intestinal inflammation. Herein, we report on CHR expression in human colon tissue from persons with UC compared with unaffected controls. Further, we evaluated effects of CHR in dextran sulfate sodium (DSS) model of colitis and assessed its effects on AAM activities and human colonic cell line functions. We report that treatment with CHR significantly ameliorates disease severity and inhibits intestinal inflammation.

## Materials and Methods

### Human Subjects

Patients who diagnosed with active UC and persons with no IBD were recruited from the University of Manitoba IBD Clinical and Research Centre. Endoscopic biopsies obtained from 10 patients with active UC and 10 healthy individuals. Patients were between 27 and 55 years and with a mean age of 40 years. Informed consent obtained from patients and control subjects before the study. This study approved by the University of Manitoba Health Research Ethics Board [HS14878 (E)].

### Animals

Experiments were approved by the University of Manitoba Animal Ethics Committee (Protocol # 15-010) and conducted under the Canadian guidelines for animal research. Six- to eight-week-old male C57BL/6 mice (20–25 g body weight) purchased from Charles River (Sherbrook, Canada) were maintained in the animal care facility at the University of Manitoba under a specific pathogen-free barrier facility.

### Peptides

Peptides purchased from Pepmic Co., Suzhou, China. Peptides were processed by reversed-phase high-performance liquid chromatography and mass spectrometry. CHR peptide corresponds to CHR (ChgA_47–66_: RILSILRHQNLLKELQDLAL) (24–26, 28, 34, 35). To confirm the sequence specificity scrambled CHR peptide (sCHR, ChgA_47–66_: RARDHQQENKILLLSLILLL) was used as an internal control. The effective dose was adjusted at 2.5 mg/kg/day as reflected by previously published data related to the use of peptide for intracolonic injection (32, 33). Control groups received intracolonic injection of 1% phosphate-buffered saline (PBS).

### Acute DSS-Experimental Colitis

Intracolonic injection of CHR or sCHR or 1% PBS started 1-day before colitis induction and lasted for 5-days. The experimental design of these experiments is illustrated in Figure 3A. DSS (molecular weight, 40 kDa: MP Biomedicals, Soho, OH, USA) was added to the drinking water at a final concentration of 5% (wt/vol) for 5 days (36) to 6- to 8-week-old mice. DSS was freshly dissolved every 2 days. Controls were time-matched with mice receiving regular drinking water only. Mean DSS consumption was noted per cage each day. Weight loss, stool consistency, and bleeding were reported (37) from day 0 to day 5 during DSS treatment. Blood in the stool was evaluated using the Hemoccult II test (Beckman Coulter, Oakville, ON, Canada). Mice were sacrificed at day5. Collagen deposition and fibrosis scores were assessed as described previously (38). Samples were isolated from the splenic flexure, fixed in formalin, paraffin embedded, sectioned in 3-µm sections, and stained using Masson’s Trichrome (Sigma, Mississauga, ON, Canada). Collagen deposition and fibrosis were scored based on a published scoring system that considers collagen deposition (score 0 = no increase, score 1 = increase in the submucosa, 2 = increase in the mucosa, 3 = increase in the muscularis mucosa and its thickening, 4 = increase in the muscularis propria, and 5 = gross disorganization in the muscularis propria) and the percent involvement (score 1 = 1–25%, score 2 = 26–50%, score 3 = 51–75%, and score 4 = 76–100%) (39).

### Colonic Protein Assay

Colonic sample were homogenized mechanically in 700 µL of Tris HCl buffer containing protease inhibitors (Sigma, Mississauga, ON, Canada) then centrifuged for 30 min, and supernatants were frozen at 80°C until assay (32). Cytokines release and arginase activity measurements were performed on clarified full-thickness colon homogenates from mice and or supernatants from cell culture using enzyme-linked immunosorbent assays (ELISAs). Commercial ELISA kits for mouse IL-10, mouse IL-18, human IL-8 and human IL-18 (R&D Systems, Inc., MN, USA), and mouse arginase activity (Abnova, Walnut, CA, USA) were used.

### Macrophage Cell Culture

Peritoneal macrophages were isolated from C57BL/6 male mice as described by Mosser and Zhang (40). Isolated macrophages were cultured in 2 mL Dulbecco’s Modified Eagle’s Medium (DMEM) supplemented with 100 U/mL penicillin, 100 µg/mL streptomycin, and 10% deactivated fetal bovine serum (FBS). Cell cultures were incubated in a humidified 5% CO_2_ incubator at 37°C. The overall cell viability of the adherent cell was greater than 95%. *Ex vivo* colitic macrophages isolation; 5 days after the beginning of the DSS treatment, resident peritoneal macrophages were isolated from all groups and subjected to further analysis. *In vitro* AAM activation; peritoneal macrophages were isolated from naive male C57BL/6 mice then serum starved overnight in DMEM with low FBS (0.5%). Macrophages were washed three times with 1% PBS solution and pretreated with CHR (200 ng/mL) for 2 h and then exposed for additional 6 h to 1% PBS in medium or IL-4/IL-13 (20 ng/mL) to induce AAM (40). Cell and supernatant medium were harvested for analysis.

### Cell Line Culture

Human IEC line, Caco-2 (ATCC, Manassas, VA, USA), was cultured in 7 mL of culture medium in a T-25 culture flask. Eagle’s Minimum Essential Medium (glutamine, high glucose) supplemented with 100 U/mL penicillin, 100 µg/mL streptomycin, and 20% deactivated FBS was used. Cells were incubated in a humidified 5% CO_2_ incubator at 37°C. Caco-2 cells were detached by using 3 mL of trypsin (0.05% trypsin, 0.53 mM EDTA) and seeded at 3 × 10^5^ cells/well onto tissue culture 24-well plates. Cells were counted using a TC20™ Automated Cell Counter (Bio-Rad Laboratories, Inc., Mississauga, ON, Canada), and cell count was verified using a conventional hemocytometer cell counting method. The cell culture medium was changed every 3 days until the cells fully differentiated (80–90% confluent). For each experimental setup, three separate experiments were performed, and at least six wells per condition were assigned.

#### Lipopolysaccharides (LPSs)- and DSS-Stimulated Epithelial Cells in the Presence or Absence of CHR-Treated AAM Conditioned Medium

Caco-2 cells were seeded at 3 × 10^5^ cells/well onto tissue culture plates. Naive peritoneal macrophages were isolated from naive C57BL6 mice and polarized toward AAM (IL-4/IL-13 20 ng/mL) in the presence or absence of CHR or sCHR (100 ng/mL) for 6 h. 2 mL of AAM supernatant or naive PBS-treated macrophage supernatant were added to the Caco-2 cell line for 24 h. Then, Caco-2 cells were challenged with LPS (1 µg/mL) (*Escherichia coli* serotype 127: B8, Sigma-Aldrich, St. Louis, MO, USA) or 5% DSS for an additional 24 h (41). The potential effects of CHR-treated AAM conditioned medium on mRNA level of TJ proteins [claudin-1 (*CLDN1*), zonula occludens-1 (*ZO1*), E-cadherin (*CADH1*), and occludin (*OCLN*)] and proinflammatory cytokines IL-8 and IL-18 were investigated. Moreover, migration, proliferation, viability, and oxidative stress survivability of Caco-2 cell line were assessed as described below.

#### Direct CHR Treatment of LPS- and DSS-Stimulated Colonic Cell Line

Caco-2 cells were seeded at 3 × 10^5^ cells/well onto tissue culture plates and treated with 2 mL of medium containing CHR, sCHR (100 ng/mL) or 1% PBS for 24 h. Then cells were challenged with LPS (1 µg/mL) or 5% DSS for an additional 24 h (41). Gene expression of TJ proteins and proinflammatory cytokines IL-8 and IL-18, migration, proliferation, viability, and oxidative stress survivability of Caco-2 cell line were evaluated.

#### Colonic Cell Line Migration Assessed by Using Wound-Healing Assay

Caco-2 cells were wounded using a sterile 100-µL pipette tip dragged perpendicular to a black line drawn on the underside of the plate for reference. Images were taken at wounding (0), 12, 24, and 48 h later using an Evos FL imaging system at 4× magnification. Wound widths were determined by averaging six measurements per image. Only scratches with edges that could be captured in one frame at the time point 0 h were included for final analysis. Measurements were taken from edge to edge at the time point 0 h and compared with measurements from 12, 24, and 48 h using ImageJ (National Institutes of Health, Bethesda, MD, USA) software (42). The reported values were the difference between time point 0 and the other time points, with higher values representing increased cellular migration.

#### Colonic Cell Line Proliferation and Viability Assessed Using Cell Numbers and MTT Assay

Colonic cell line viability was studied *in vitro* by using the 3-(4, 5-dimethyl thiazolyl-2yl)-2, 5-diphenyl tetrazolium (MTT) assay. Briefly, Caco-2 cells were seeded into 96-well plates at a density of 5 × 10^5^ cells/well and serum starved for 24 h. Cells were cultured for 3 days in 200 µL of medium containing CHR (100 nmol/mL) or sCHR (100 nmol/mL). Negative controls received 200 µL of medium, containing vehicle only (1% PBS). After 72 h, the media was aspirated and cells quantified by MTT assay (Trevigen Inc., Gaithersburg, MD, USA) according to the manufacturer’s instructions. The plates were quantified using a microplate spectrophotometer (Molecular Devices, Sunnyvale, CA, USA) at a wavelength of 570 nm.

#### Colonic Cell Line Survival Using an Oxidative Stress Assay

2 mL of 200 mmol/L of H_2_O_2_ in PBS were given to the Caco-2 cells for 30 min. Trypan blue staining was performed to count viable cells.

### Quantitative Real-time Reverse-Transcription Polymerase Chain Reaction (PCR)

Total RNA was extracted using TRIzol™ Plus RNA Purification Kit (Life Technologies, NY, USA) and reverse transcribed using SuperScript VILO cDNA Synthesis Master Mix (Invitrogen, NY, USA). A real-time quantitative PCR was used to quantify gene expression in a Roche light cycler 96 Real-Time System using Power SYBR green master mix (Life Technologies, Burlington, ON, Canada). Differences in the threshold cycle (ΔCt) number between the target genes and mouse eukaryotic elongation factor 2 (*Eef2*) and human TATA-box binding protein (*TBP*) (optimal reference genes) (43–45) were used to normalize expression. Human and mice primers sequences for the genes that encode cytokines, TJ proteins and IECs markers are provided in Tables 1 and 2.

**Table 1:** Human primers sequences.

Gene name	Forward	Reverse
*IL-10*	GACTTTAAGGGTTACCTGGGTTG	TCACATGCGCCTTGATGTCTG
*MR*	GGAGTGATGGTTCTCCTGTTTC	CCTTTCAGCTCACCACAGTATT
*CD1B*	ACTCAGGAAATCCAATCCTCCTA	ATAGCAGGCTGTGAGCTACAT
*OCLDN*	ACAAGCGGTTTTATCCAGAGTC	GTCATCCACAGGCGAAGTTAAT
*TBP*	CCCGAAACGCCGAATATAATCC	AATCAGTGCCGTGGTTCGTG
*CLDN1*	AGGTGCTATCTGTTCAGTGATG	TGGCTGACTTTCCTTGTGTAG
*CADH1*	CTTCTGCTGATCCTGTCTGATG	TGCTGTGAAGGGAGATGTATTG
*ZO1*	CCAGCCTGCTAAACCTACTAAA	ATCTCTTGCTGCCAAACTATCT
*COL1A2*	GAGCGGTAACAAGGGTGAGC	CTTCCCCATTAGGGCCTCTC
*IL-8*	ACTGAGAGTGATTGAGAGTGGAC	AACCCTCTGCACCCAGTTTTC
*IL-18*	GCGTCACTACACTCAGCTAAT	GCGTCACTACACTCAGCTAAT
*CHR* (*CHGA Exon-IV*)	TCATTGCAGATGAACGGAT	TTGGAGAGCGAGGTCTT

**Table 2:** Mouse primers sequences.

Gene	Forward	Reverse
*Il10*	GCTCTTACTGACTGGCATGAG	CGCAGCTCTAGGAGCATGTG
*Arg1*	TTGGGTGGATGCTCACACTG	GTACACGATGTCTTTGGCAGA
*Il18*	GACTCTTGCGTCAACTTCAAGG	CAGGCTGTCTTTTGTCAACGA
*Ym1*	CAGGTCTGGCAATTCTTCTGAA	GTCTTGCTCATGTGTGTAAGTGA
*Fizz1*	AAGCCTACACTGTGTTTCCTTTT	GCTTCCTTGATCCTTTGATCCAC
*Col1a2*	GGTGAGCCTGGTCAAACGG	ACTGTGTCCTTTCACGCCTTT
*Eef2*	TGTCAGTCATCGCCCATGTG	CATCCTTGCGAGTGTCAGTGA
*Ocldn*	TTGAAAGTCCACCTCCTTACAGA	CCGGATAAAAAGAGTACGCTGG
*Cldn1*	GGGGACAACATCGTGACCG	AGGAGTCGAAGACTTTGCACT
*Zo1*	GCCGCTAAGAGCACAGCAA	TCCCCACTCTGAAAATGAGGA
*Cadh1*	CATCCCAGAACCTCGAAACA	TGGGTTAGCTCAGCAGTAAAG

### Data Analysis

Group comparisons were determined using unpaired Mann–Whitney *U* test, and one- and two-way ANOVA followed by a *post hoc* test when appropriate. Spearman’s correlation test was used. *P* values (two-tailed) below 0.05 were considered as significant. Data are presented as mean ± SEM and statistics were analyzed using GraphPad Prism software (version 6; GraphPad Software, Inc., La Jolla, CA, USA).

## Results

### CHR Correlates Positively with AAM and Gene Expression of TJ Proteins and Negatively with IL-8, IL-18, and Collagen Gene Expression in Patients with Active UC

**Figure 1:**
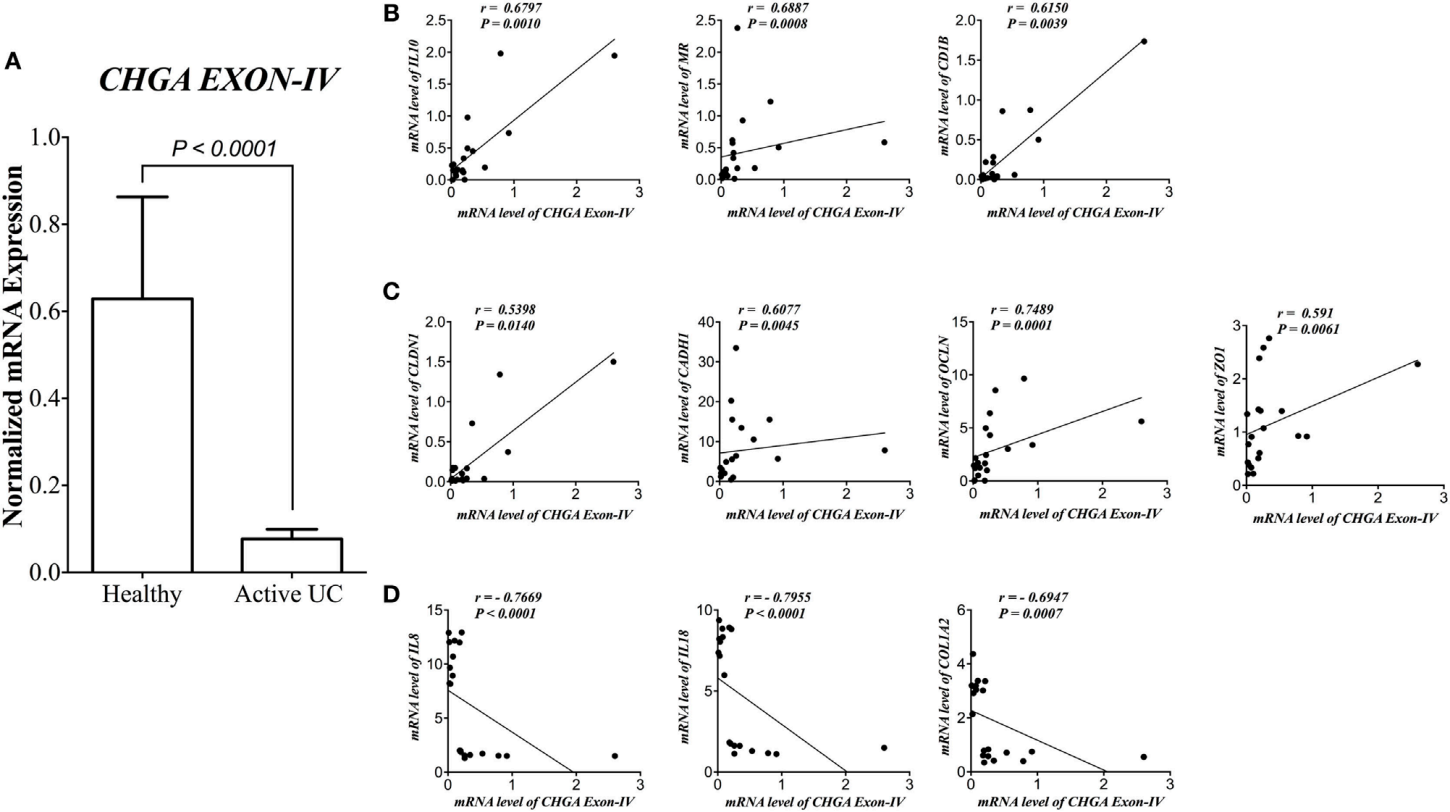
Chromofungin (CHR) correlates positively with alternatively activated macrophages (AAMs) and gene expression of tight junction (TJ) proteins and negatively with interleukin (*IL*)*-8, IL-18* and collagen gene expression in patients with active ulcerative colitis (UC). mRNA levels of **(A)** CHR (*CHGA Exon-IV*) in human active UC (*n* = 10), and healthy individual as control (*n* = 10) and its correlation with mRNA levels of **(B)** AAM markers [*IL-10*, mannose receptor (*MR*), cluster of differentiation 1B (*CD1B*)], **(C)** gene expression of tight junction (TJ) proteins, Claudin-1 (*CLDN1*), zonula occludens-1 (*ZO1*), E-cadherin (*CDH1*), and occludin (*OCLN*), **(D)** and *IL-8, IL-18*, and collagen (*COL1A2*). Mann–Whitney test and Spearman’s correlation were used to analyze the data. Two tails significance level adjusted at 0.05.

First, we assessed the relationship between CHR and human pathophysiological markers implicated in IBD. mRNA level of *CHR* was significantly reduced (*P* < 0.0001) in biopsies from subjects with active UC when compared with healthy controls (Figure 1A). *CHR* mRNA expression demonstrated a strong positive correlation with *IL-10*, mannose receptor (*CD206, MR*), cluster of differentiation 1B (*CD1B*, Figure 1B), and *CLDN1, CADH1, OCLN, ZO1* (Figure 1C). Conversely, *CHR* mRNA expression revealed a significant negative correlation with *IL-8, IL-18*, and *COL12A* (Figure 1D).

### AAM Markers Correlates Positively with Gene Expression of TJ Proteins and Negatively with IL-8, IL-18, and Collagen Gene Expression in Patients with Active UC

**Figure 2:**
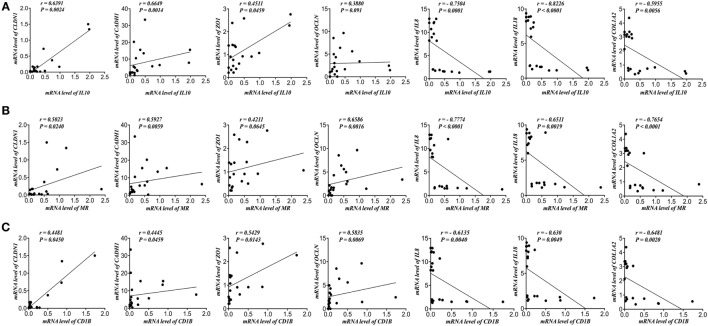
Alternatively activated macrophages (AAMs) markers correlate positively with gene expression of tight junction (TJ) proteins and negatively with interleukin (*IL*)*-8, IL-18*, and collagen gene expression in patients with active ulcerative colitis (UC). Correlation analysis of mRNA levels of **(A)** *IL-10*, **(B)** mannose receptor (*MR*), and **(C)** Cluster of Differentiation 1B (*CD1B*) with mRNA levels of Claudin-1 (*CLDN1*), zonula occludens-1 (*ZO1*), E-cadherin (*CDH1*), occludin (*OCLN*), *IL*-8, *IL-18*, and collagen (*COL1A2*). Correlation analysis: Spearman’s correlation and significance level adjusted at 0.05.

Next, we investigated the link between the genes expression of AAM markers and TJ proteins. *IL-10* mRNA expression correlated positively with *CLDN1, CADH1, ZO1*, and *OCLN* and negatively with *IL-8, IL-18*, and *COL12A* (Figure 2A). Also, *MR* correlated positively with *CLDN1, CADH1, ZO1*, and *OCLN* and negatively with *IL-8, IL-18*, and *COL12A* (Figure 2B). Moreover, *CD1B* correlated positively with *CLDN1, CADH1, ZO1*, and *OCLN* and negatively with *IL-8, IL-18*, and *COL12A* (Figure 2C).

### CHR Attenuates the Onset and Severity of DSS-Induced Colitis

**Figure 3:**
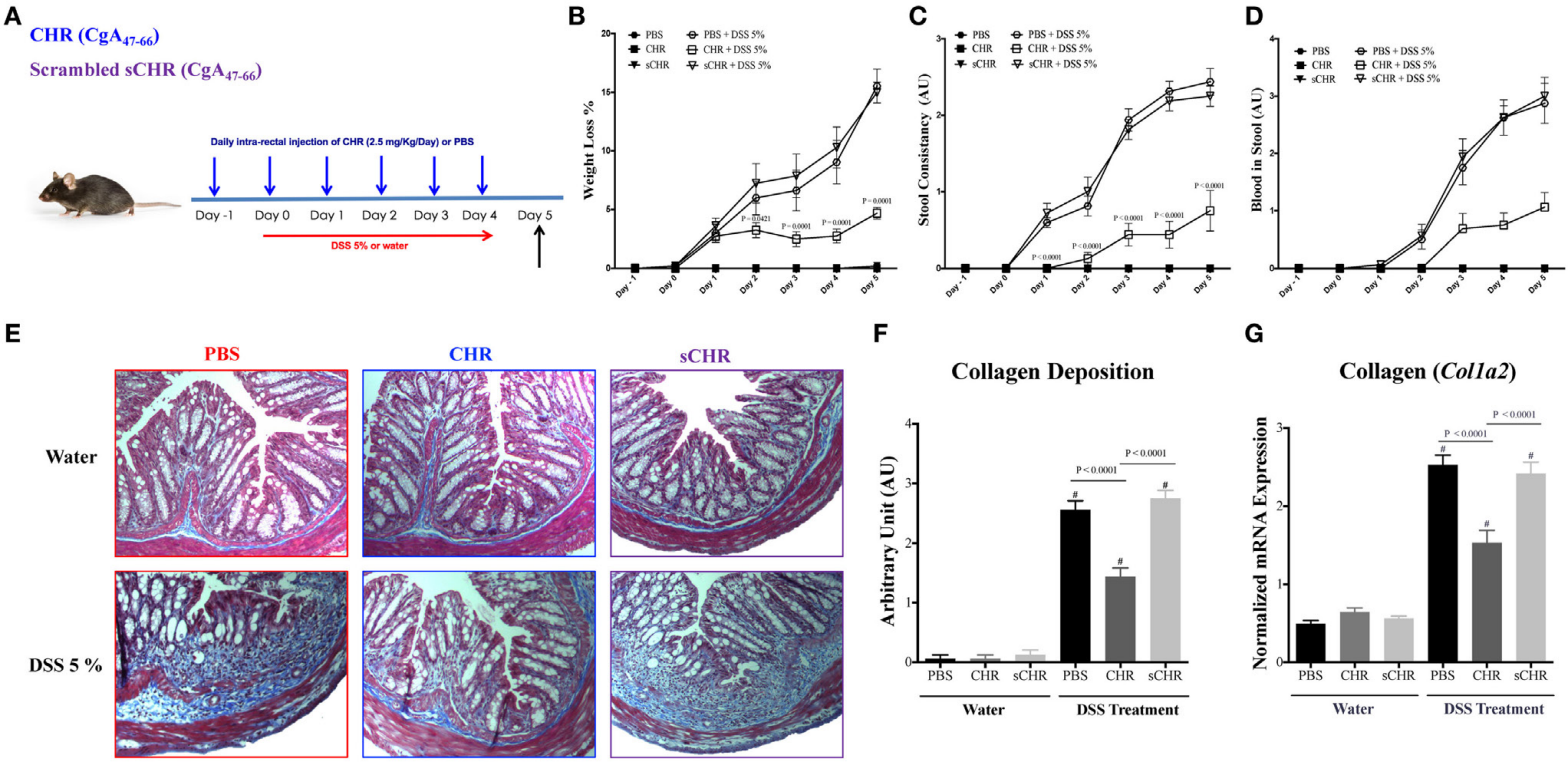
Chromofungin (CHR) attenuates colonic inflammation and reduces colonic collagen deposition in dextran sulfate sodium (DSS)-induced colitis in mice. **(A)** Experimental design of peptides treatment and DSS-induced colitis. Mice were received 5% DSS for 5 days. Mice received an intracolonic injection of CHR peptide (2.5 mg/kg/day) or scrambled CHR peptide (2.5 mg/kg/day) or vehicle phosphate-buffered saline 1% (control) starting 1 day before DSS treatment. Disease onset and severity (from day 0 to day 5) represented by **(B)** percentage of body weight change of different groups, **(C)** stool consistency, and **(D)** blood in stool. Colonic collagen deposition scores were quantified by **(E,F)** Masson Trichrome staining for collagen in colonic tissues, whereas collagen stained in blue with a red background. **(G)** Quantitative real-time reverse-transcription polymerase chain reaction (RT-PCR) of collagen col1a2 mRNA expression in colonic tissues of mice. Two-way repeated measures or one-way ANOVA followed by multiple comparison tests. Each value represents the mean ± SEM, *n* = 8–10 mice/group. ^#^ refers to significance compared to control groups. Each experiment was repeated at least three times.

To decipher the functional consequences of exogenous CHR administration, a mouse model of colitis was used. Preventive intracolonic administration of CHR to DSS-treated mice decreased significantly (*P* ≤ 0.0001) the clinical signs of colitis, represented by the weight loss percentage, stool consistency and stool bleeding (Figures 3B–D). In colitic mice, intracolonic administration of CHR significantly reduced the collagen deposition and fibrosis scores (Figures 3E,F). Moreover, DSS administration increased *Col1a2* mRNA colonic expression (Figure 3G) and treatment with CHR decreased it significantly (Figure 3G). Administration of the sCHR peptide did not modify the markers studied.

### CHR Decreases IL-18 Release and Regulates Colonic Gene Expression of TJ Proteins in DSS-Induced Colitis

**Figure 4:**
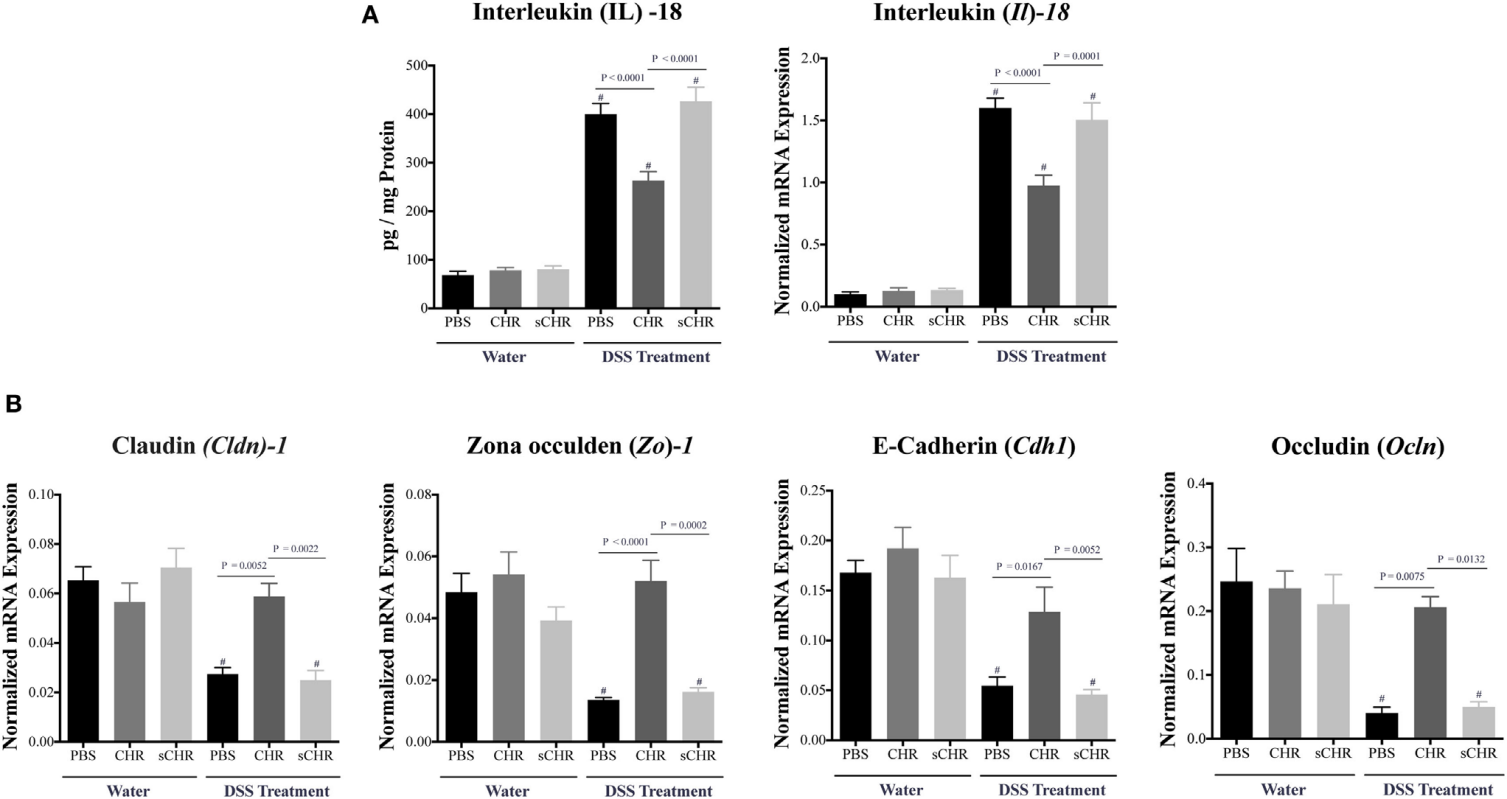
Chromofungin (CHR) decreases interleukin (IL)-18 release and maintains colonic gene expression of tight junction (TJ) proteins in dextran sulfate sodium (DSS)-induced colitis. Treatments [CHR or sCHR (2.5 mg/kg/day) or 1% phosphate-buffered saline (PBS)] started 1 day prior to colitis induction. **(A)** Colonic protein and mRNA expression levels of *Il-18*. **(B)** Colonic mRNA levels of TJ proteins [claudin-1 (*Cldn1*), zonula occludens-1 (*Zo1*), E-cadherin (*Cdh1*) and occludin (*Ocln*)]. One-way ANOVA followed by multiple comparison tests. Each value represents the mean ± SEM, *n* = 8–10 mice/group. ^#^ refers to significance compared to control groups. Each experiment was repeated at least three times.

Tight junction proteins and IL-18 play critical roles during the progression of IBD (12, 46). Compared with non-colitic mice, a significant decrease in *Cldn1, Zo1, Cdh1*, and *Ocln* colonic mRNA levels was detected in DSS-treated mice (Figure 4B), however, CHR treatment abolished this effect (Figures 4A,B). We also observed that DSS treatment elevated colonic protein and mRNA expression levels of IL-18 (Figure 4A) which was significantly decreased when mice were treated with CHR (Figure 4A). Administration of the sCHR peptide neither modified the control conditions nor the deleterious effect of the DSS treatment.

### CHR Increases AAM Polarization and Increases Anti-inflammatory Mediators in DSS-Induced Colitis

**Figure 5:**
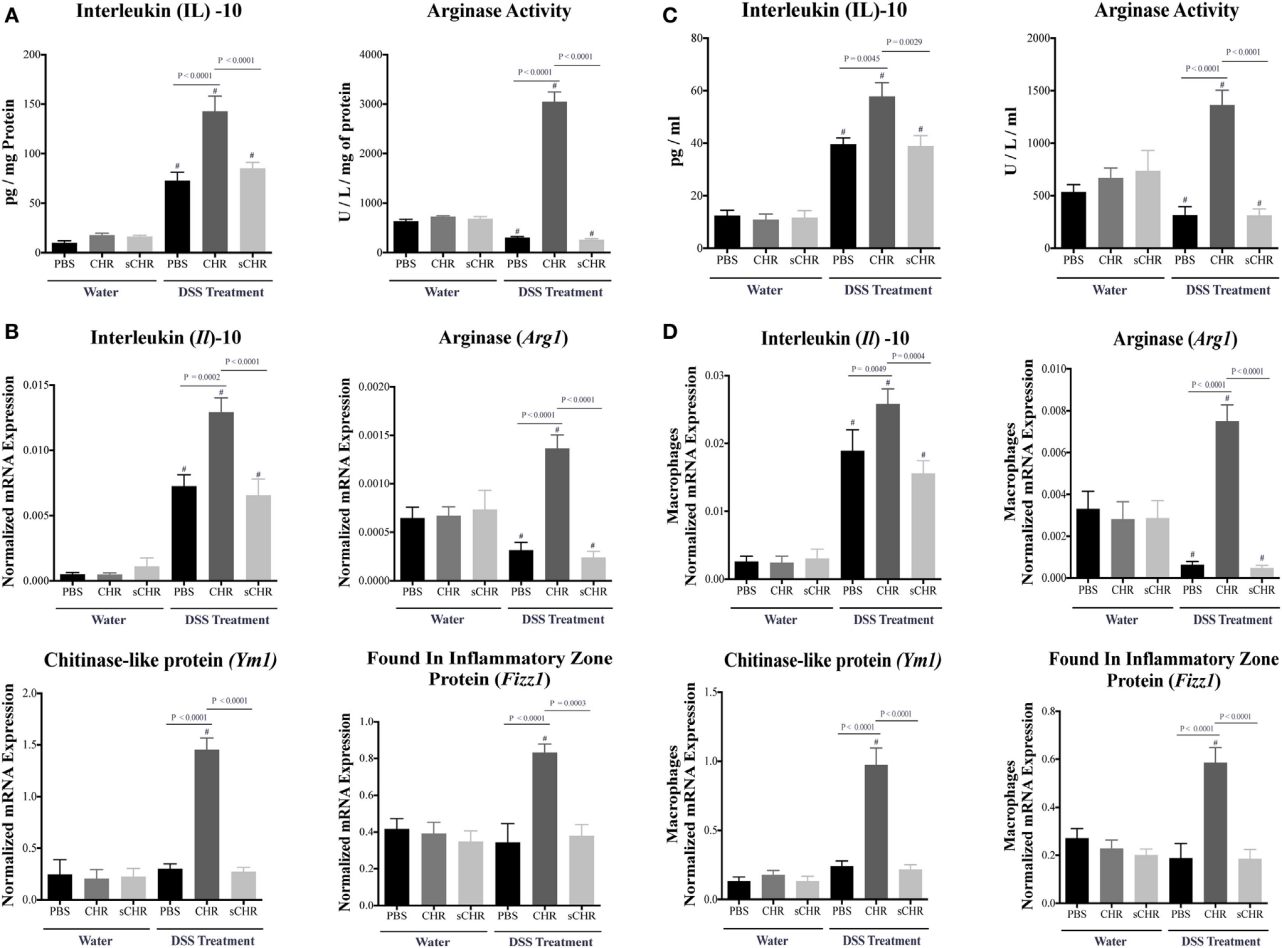
Chromofungin (CHR) upregulates the activity of alternatively activated macrophages (AAM) in dextran sulfate sodium (DSS)-induced colitis. Preventive treatments of CHR or sCHR or 1% 1% phosphate-buffered saline (PBS) were started 1 day prior to colitis induction. **(A)** Colonic protein levels of interleukin (IL)-10 and arginase activity and **(B)** colonic mRNA levels of AAM markers [*Il10*, arginase (*Arg1*), *Ym1* Chitinase-like protein (*Ym1*), and found in inflammatory zone protein (*Fizz1*)]. Peritoneal macrophages isolated from all mice groups; **(C)** protein levels of IL-10 and arginase activity, and **(D)** mRNA levels of AAM markers (*Il10, arg1, Fizz1*, and *Ym1*) in the peritoneal macrophages. One-way ANOVA followed by multiple comparison tests. Each value represents the mean ± SEM, *n* = 8–10 mice/group. ^#^ refers to significance compared to control groups. Each experiment was repeated at least three times.

Alternatively activated macrophage plays a significant role in colonic tissue-repair through the high production of IL-10, arginase, and other extracellular molecules (47, 48). Therefore, to further determine the role of CHR in modulating immune cells during the development of colitis, colonic AAM mediators and markers were investigated. Colitic CHR-treated mice displayed an increase in IL-10 and Arginase activity (Figure 5A), moreover, mRNA expression of *Il10*, arginase (*Arg1*), *Ym1* Chitinase-like protein (*Ym1*), and found in inflammatory zone protein (*Fizz1*) were significantly upregulated (Figure 5B). To confirm this effect, we next investigated the role of CHR in peritoneal macrophage isolated from colitic mice. Measurement of AAM mediators and markers revealed an increase in IL-10 and arginase activity in response to CHR (Figure 5C), along with increased mRNA expression of *Il10, Arg1, Ym1, and Fizz1* (Figure 5D), when compared to macrophages isolated from the colitic PBS-treated group. Administration of the sCHR peptide neither modified the control conditions nor the deleterious effect of the DSS treatment.

### CHR Enhances the Polarization of Naive Peritoneal AAM

**Figure 6:**
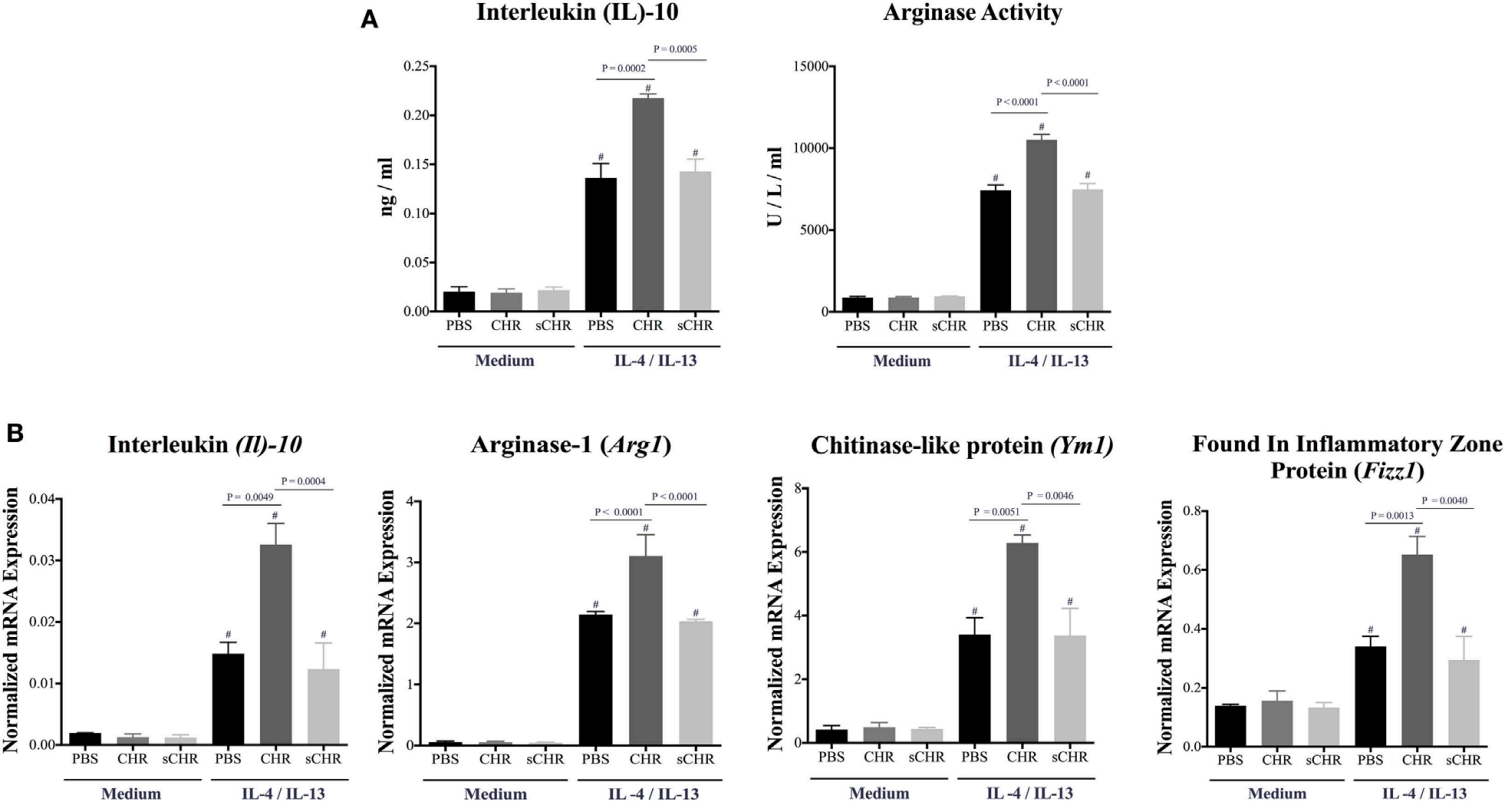
Chromofungin (CHR) enhances the polarization of alternatively activated macrophages (AAM) *in vitro*. Peritoneal macrophages collected from naive C57BL6 mice and pretreated with CHR (200 ng/mL) for 2 h then stimulated by interleukin (IL)-4/IL-13 (20 ng/mL) for 6 h. **(A)** Protein levels of IL-10 and arginase activity and **(B)** mRNA levels of AAM markers [*Il10*, arginase (*Arg1*), *Ym1* chitinase-like protein (*Ym1*), and found in inflammatory zone protein (*Fizz1*)]. One-way ANOVA followed by multiple comparison tests. Each value represents the mean ± SEM, *n* = 3–5/group. ^#^ refers to significance compared to control groups, Each experiment repeated at least three times.

Considering the effect of other CHGA-derived peptides on macrophages and their contribution to macrophages polarization (32, 33, 49–52), we reasoned that CHR might be involved in AAM polarization. To determine whether CHR can directly affect the polarization of AAM, peritoneal macrophage of naive C57BL6 mice were isolated and pretreated with CHR and polarized toward AAM using IL-4/IL-13. CHR pretreatment increased mRNA expression levels of AAM markers, *Il10, Arg1, Fizz1, and Ym1*, and the release of IL-10 and Arginase activity (Figures 6A,B). Administration of the sCHR peptide neither modified the control conditions nor the deleterious effect of the IL-4/IL-13 treatment.

### CHR-Treated AAM Conditioned Medium Maintains Gene Expression of TJ Proteins and Decreases IL-8 and IL-18 Release in LPS- and DSS-Stimulated Colonic Epithelial Cells

**Figure 7:**
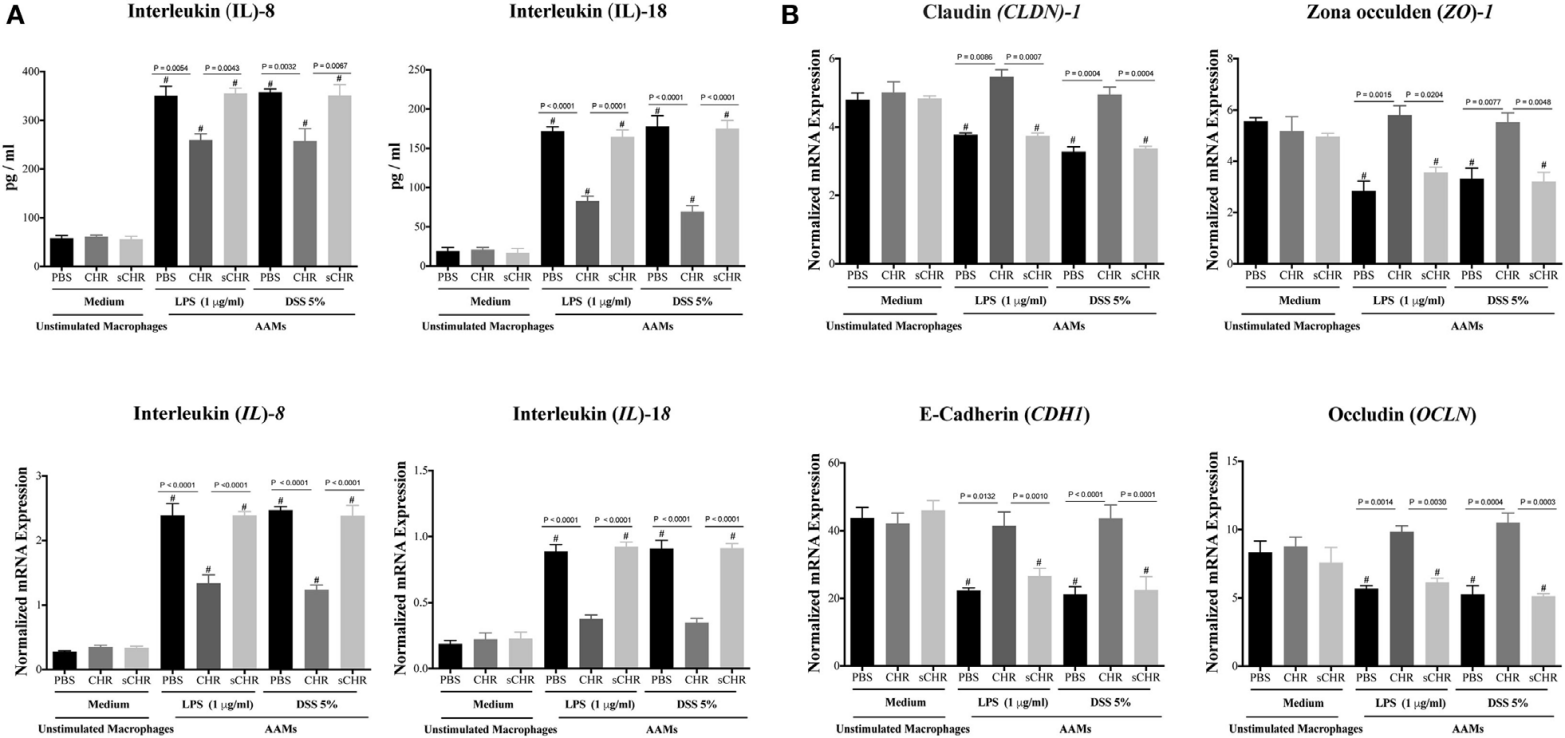
Chromofungin (CHR) indirectly maintains gene expression of tight junction (TJ) proteins and decreases interleukin (IL)-8 and IL-18 release from lipopolysaccharide (LPS)- and dextran sulfate sodium (DSS)-stimulated colonic cell line through alternatively activated macrophages (AAM) conditioned medium. Peritoneal macrophages collected from naive C57BL6 mice and pretreated with CHR (200 ng/mL) for 2 h then stimulated by IL-4/IL-13 (20 ng/mL) for 6 h. Caco-2 cells were cultured in 2 mL supernatants of 1% phosphate-buffered saline (PBS) or CHR (100 nmol/mL) or sCHR (100 nmol/mL) treated AAM conditioned medium for 24 h, then challenged with LPS (1 µg/mL) or 5% DSS for 24 h. Cells and supernatants harvested for analysis. **(A)** IL-8 and IL-18. **(B)** Colonic mRNA levels of TJ proteins [claudin-1 (*CLDN1*), zonula occludens-1 (*ZO1*), E-cadherin (*CDH1*), occludin (*OCLN*)]. One-way ANOVA followed by multiple comparison tests. Data represent mean ± SEM (*n* = 6). ^#^ refers to significance compared to control groups. Each experiment repeated at least three times.

The human Caco-2 IEC system has been commonly used as an *in vitro* model of the intestinal epithelium (53–55). Also, caco-2 cells have been used as *in vitro* model of IBD for potential drug testing and screening (56–60). Therefore, culture studies were performed using Caco-2 epithelial cells and AAM conditioned medium to assess whether CHR-treated AAM conditioned medium could regulate the expression and the release of IL-8 and IL-18 and the gene expression of TJ proteins in a human colonic cell line following LPS or DSS-induced injury. Exposing Caco-2 cells to LPS (1 µg/mL) or 5% DSS for 24 h induced a significant increase of IL-8 and IL-18 release (Figure 7A) and a substantial downregulation of mRNA expression levels of *Cldn1, Zo1, Cdh1*, and *Ocln* (Figure 7B). Conversely, the presence of CHR-treated AAM conditioned medium maintained barrier restitution by suppressing IL-8 and IL-18 release (Figure 7A) and by maintaining the mRNA expression of *CLDN1, ZO1, CADH1*, and *OCLN* (Figure 7B). Administration of the sCHR peptide neither modified the control conditions nor the deleterious effect induced by LPS or DSS treatments.

### CHR Maintains Gene Expression of TJ Proteins and Decreases IL-8 and IL-18 Release in LPS- and DSS-Stimulated Colonic Epithelial Cell Line

**Figure 8:**
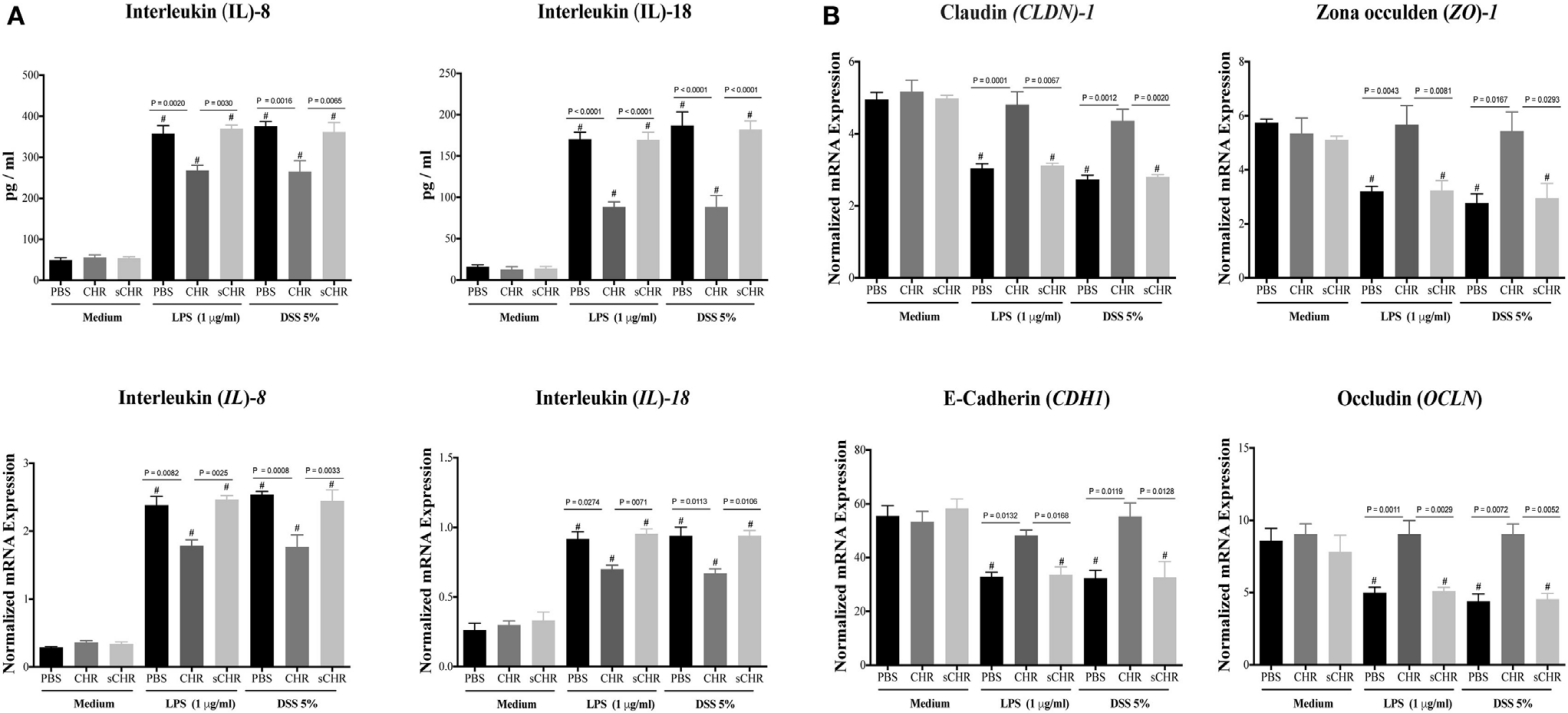
Chromofungin (CHR) directly maintains gene expression of tight junction (TJ) proteins and decreases interleukin (IL)-8 and IL-18 release from lipopolysaccharide (LPS)- and dextran sulfate sodium (DSS)-stimulated colonic cell line. Caco-2 cells were treated with 1% phosphate-buffered saline (PBS) or CHR (100 nmol/mL) or sCHR (100 nmol/mL) in medium for 24 h then challenged with LPS (1 µg/mL) or 5% DSS for additional 24 h. **(A)** IL-8 and IL-18. **(B)** Colonic mRNA levels of TJ proteins [claudin-1 (*CLDN1*), zonula occludens-1 (*ZO1*), E-cadherin (*CDH1*), occludin (*OCLN*)]. One-way ANOVA was used to analyze the data followed by multiple comparison tests. Data represent mean ± SEM (*n* = 6). ^#^ refers to significance compared to control groups. Each experiment repeated at least three times.

Furthermore, we investigated whether CHR could have a direct effect on the expression/release of IL-8 and IL-18 and the gene expression of TJ proteins following LPS or DSS-induced injury. CHR treatment maintained the epithelial homeostasis by suppressing IL-8 and IL-18 release (Figure 8A) and by maintaining the gene expression of *CLDN1, ZO1, CADH1, OCLN* (Figure 8B). Moreover, in the absence of stimuli, CHR treatment did not show any significant effects on IL-8 and IL-18 release or mRNA levels of TJ proteins (Figures 8A,B). Administration of the sCHR peptide neither modified the control conditions nor the deleterious effect of induced by LPS or DSS treatments.

### CHR-Treated AAM Conditioned Medium Promotes Epithelial Migration, Proliferation, Viability, and Oxidative Stress Viability

**Figure 9:**
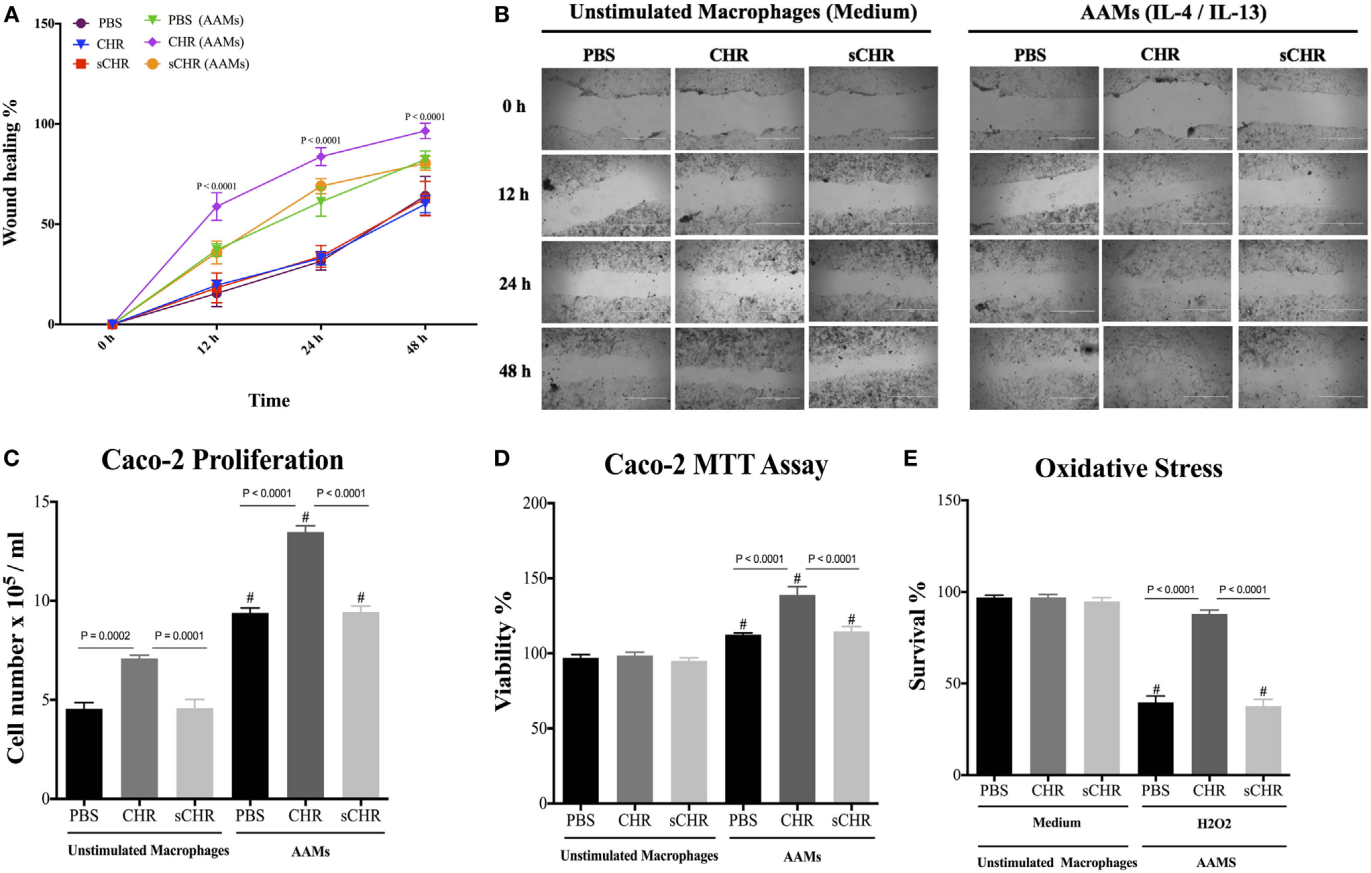
Chromofungin (CHR) indirectly induces migration, proliferation, viability, and oxidative stress survivability of colonic cell line through alternatively activated macrophages (AAM)-conditioned medium. Macrophages were treated with CHR (200 ng/2 h) then stimulated by interleukin (IL)-4/IL-13 (20 ng/mL) to promote AAM for 6 h and supernatants were collected. Caco-2 cells were cultured in 2 mL of supernatants of 1% phosphate-buffered saline (PBS) or CHR (100 nmol/mL) or sCHR (100 nmol/mL) treated AAM conditioned medium. **(A,B)** Epithelial cell migration assessed by the wound healing assay, **(C)** intestinal epithelial cell proliferation, **(D)** epithelial cell viability assessed by the 3-(4, 5-dimethyl thiazolyl-2yl)-2, 5-diphenyl tetrazolium (MTT) assay, and **(E)** epithelial cells oxidative stress assay show survival data from cultures treated with normal medium (control) or 200 mmol/L H_2_O_2_. Two-way or one-way ANOVA was used to analyze the data followed by multiple comparison tests. Data represent mean ± SEM (*n* = 6). ^#^ refers to significance compared to control groups. Each experiment was repeated at least three times.

The appropriate activation of AAM is crucial for tissue repair (48), and IBD involves functional impairment of IECs, associated with infiltration of macrophages in the lamina propria (6, 61, 62). Macrophages can mediate protective effects via a variety of mechanisms, including maintenance or reshaping of the epithelial homeostasis through cell proliferation and migration, and by promoting resistance to epithelial apoptosis induced by oxidative stress (5, 63). Therefore, we investigated the potential consequences of CHR-treated AAM conditioned medium on the functions of colonic epithelial cells using Caco-2 cells. In the presence of CHR-treated AAM conditioned medium, migration, viability, and proliferation of the epithelial cells increased (Figures 9A–D). Administration of the sCHR peptide did not have any effect on cell proliferation or migration. Furthermore, oxidative stress is a feature of intestinal inflammation and initiates epithelial apoptosis (17, 64). Therefore, Caco-2 cells were exposed to the free radical donor, H_2_O_2_, and cell survival was assessed. H_2_O_2_ caused a significant reduction in cell survival compared with untreated cells, and cell survival in H_2_O_2_-treated cultures significantly improved in the presence of CHR-treated AAM-conditioned medium (Figure 9E). Administration of the sCHR peptide neither modified the control conditions nor the deleterious effect of induced by H2O2.

### CHR Enhances Epithelial Migration, Proliferation, Viability, and Oxidative Stress Viability

**Figure 10:**
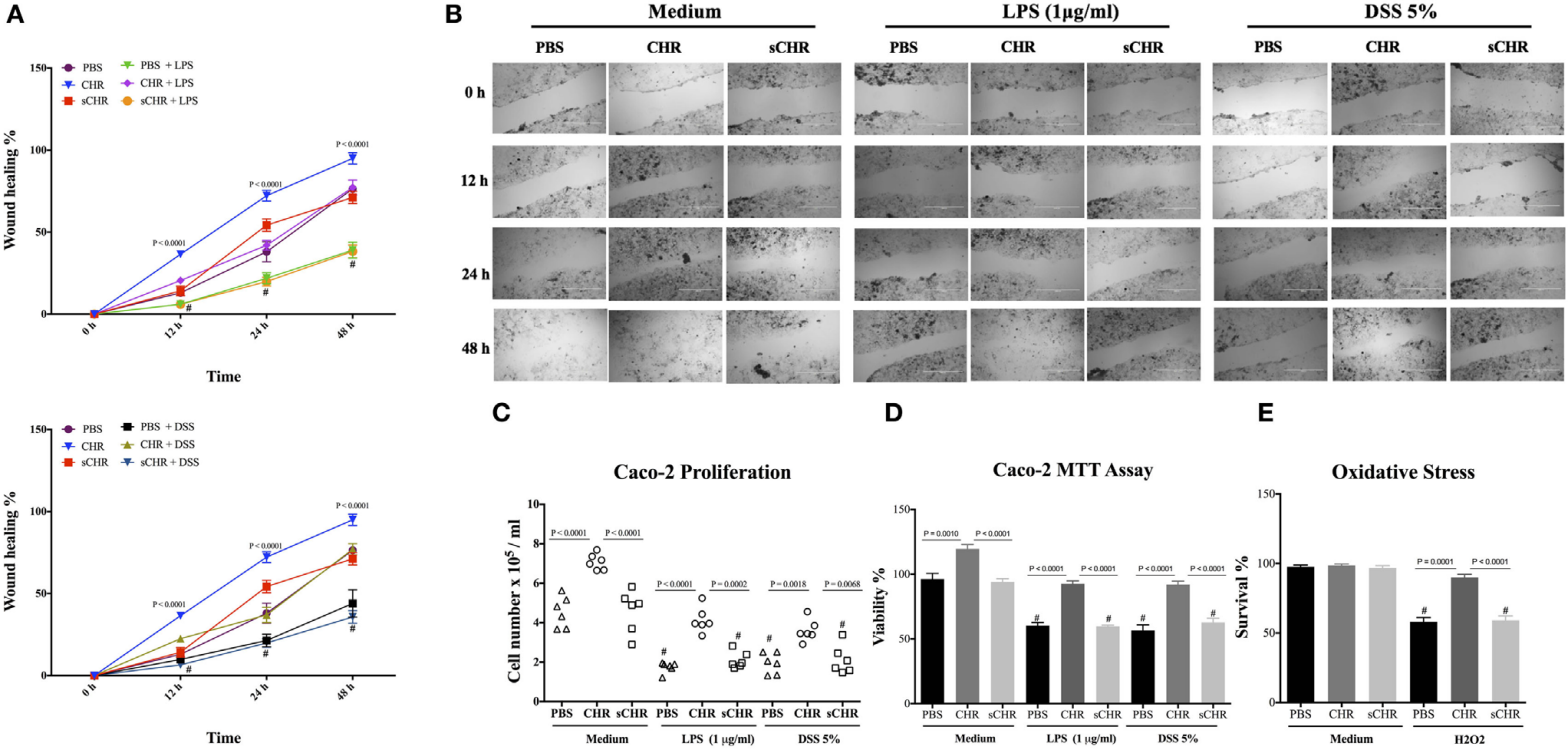
Chromofungin (CHR) directly enhances migration, proliferation, and viability of colonic cell line. Caco-2 cells were pre-treated with 1% phosphate-buffered saline (PBS) or CHR (100 nmol/mL) or sCHR (100 nmol/mL) for 24 h, then challenged with lipopolysaccharide (LPS) (1 µg/mL) or 5% dextran sulfate sodium (DSS) for additional 24 h. **(A,B)** Epithelial cell migration assessed by the wound healing assay, **(C)** intestinal epithelial cell proliferation, **(D)** epithelial cell viability assessed by the 3-(4, 5-dimethyl thiazolyl-2yl)-2, 5-diphenyl tetrazolium (MTT) assay, and **(E)** epithelial cells oxidative stress assay shows survival data from cultures treated with normal medium (control) or 200 mmol/L H_2_O_2_. Two-way or one-way ANOVA was used to analyze the data followed by multiple comparison tests. Data represent mean ± SEM (*n* = 6). ^#^ refers to significance compared to control groups. Each experiment was repeated at least three times.

Finally, we investigated the direct interaction between human cell line and CHR in LPS- and DSS-stimulated cells. Exposing Caco-2 cells to LPS (1 µg/mL) or 5% DSS for 24 h led to a significant decrease in the cell migration, cell proliferation and viability (Figures 10A–D) and exogenous CHR treatment restored these properties (Figures 10A–D). Surprisingly, in the absence of stimuli, CHR alone induced a significant increase in migration, viability, and proliferation of the cells (Figures 10A–D). H_2_O_2_ caused a significant reduction in cell survival compared with untreated cells, and treatment with CHR significantly improved it (Figure 10E). Administration of the sCHR peptide neither modified the control conditions nor the effect on cell proliferation or migration and the deleterious effect of induced by H2O2.

## Discussion

This study, for the first time, shows possible novel mechanisms by which CHR ameliorated intestinal inflammation by regulating IECs homeostasis and enhancing the activity of AAM in preclinical models. In patients with active UC, CHR showed a positive correlation with AAM markers and gene expression of TJ proteins and a negative correlation with IL-8, IL-18, and collagen gene expression. Experimentally, CHR treatment reduced the onset and severity of colitis, decreased colonic collagen deposition, promoted AAM mediators, and ultimately maintained the homeostasis of IECs during the development of DSS-induced colitis. Although CHR alone had no apparent effect on the AAM, CHR significantly expanded the polarization of AAM in the presence of IL-4/IL-13. Moreover, CHR indirectly and directly regulated colonic gene expression of TJ proteins, decreased IL-8 and IL-18 release in LPS- and DSS-stimulated human colonic epithelial cell line, and exhibited a protective effect in regulating epithelial cell migration, proliferation, viability, and oxidative stress survivability. Taken together, these findings extend the influence of CHGA-derived peptides to intestinal inflammation.

A complex network of events at molecular, cellular, and tissue levels underlie inflammation and remodeling that are tightly regulated by various mediators and mechanisms and that eventually contribute to the development of IBD. One of these molecules is the CHGA and its derived peptides, which, have emerged as an essential axis in immune cells migration and immune responses in IBD (32, 33). Recently, it has been described that CHR can affect neutrophils (25, 65). In our study, we demonstrated a positive correlation between the expression of CHR and AAM markers in patients with active UC. Experimentally, intracolonic administration of CHR reduced colitis severity through the production of IL-10 and arginase activities and the promotion of AAM-associated gene expression (*Ym1, Fizz1*) in the colonic mucosa and peritoneal macrophages. Although peritoneal macrophages are present at a distance from the mucosal inflammatory site, several studies have implicated these cells in the progression of colitis and in the unbalanced proinflammatory and anti-inflammatory axis (66–68). Previously, we reported that intracolonic administration of CHGA-derived peptides reduced the clinical sequelae of colitis and modulated the functional activity of peritoneal macrophages (32, 69). In addition to that, CHR can penetrate the cells and interfere with some intracellular pathways (70–72). It is therefore possible that intrarectal administration not only has local effects on the colonic mucosa but may also exert effects in the surrounding and adjacent tissues and cavities. Activation of anti-inflammatory AAM by stimulatory signals (IL-4, IL-13, or TGFβ1) (40) can restrain the proinflammatory immune responses through the release of anti-inflammatory molecules and various components affecting the extracellular matrix and tissue repair (8). Over the past decade, several studies have demonstrated an excessive production of proinflammatory Th1- and Th17-related cytokines (73) and a reduced AAM number in the gut of patients with IBD (9) and experimental studies confirmed these observations. DSS-induced colitis is mainly driven by an activation of CAMs and treatments with drugs interfering with their proinflammatory function result in amelioration of the intestinal inflammation (32). Conversely, it has been demonstrated that AAM can decrease the onset and severity of murine colitis (9). AAM not only protect against colitis directly but also can support the directionally concordant expansion of the Treg/Th17 cell axis associated with a restoration of the gastrointestinal immune tolerance and the repair of mucosal injuries (62). For example, Lupeol™ can mitigate intestinal inflammation by inducing and increasing survival from lethal DSS-induced colitis by upregulating AAM-related genes and downregulating CAMs-related genes (74). Furthermore, worm infections have been associated with a reduced progression of colitis through the increase of IL-4/IL-13 and the upregulation of AAM (9).

Intestinal injury and inflammation can induce excessive transmural extracellular matrix collagen deposition accompanied by an alteration of normal tissue architecture leading ultimately to fibrosis (39). Here, we reported that CHR negatively correlated with collagen expression in patients with active UC and that exogenous CHR treatment decreased significantly colonic collagen expression and deposition and protected against DSS-induced colitis. In that context it has been described that the arginase activity by murine AAM can facilitate the assembly of proline, which is critical for collagen production (75). AAM–fibroblast interaction is imperative for wound healing, but a dysregulated interaction can result in fibrosis and possibly stricture formation in the gastrointestinal tract. Although the role of AAM in the pathophysiology of fibrosis is not clear, some studies suggest that AAM display a profibrotic profile and stimulate collagen deposition (75–79), conversely other reports demonstrate that AAM can protect against fibrosis (80, 81). Here, CHR displayed a unique feature by reducing colitis severity and maintaining the IECs homeostasis without promoting collagen deposition and fibrosis. Therefore, it can be postulated that the role of AAM in collagen synthesis and deposition can be influenced by the surrounding microenvironment and the type of tissue. Our data confirm the non-deleterious effect of AAM activation in the context of colonic inflammation as demonstrated previously by other groups (9, 82).

Tight junction-deficient mouse models revealed pathophysiologic features of mucosal inflammation compatible with human UC (83). As intestinal epithelial barrier is regulated by TJ proteins (12) and as regulation of TJ proteins is correlated with intestinal inflammation (13, 14), we quantified the gene expression of TJ proteins in colonic tissue. In patients with active UC, we demonstrated a strong positive correlation between CHR and gene expression of TJ proteins and AAM. In our animal model, we showed that CHR treatment ameliorated the disease severity by maintaining colonic gene expression of TJ proteins and enhancing polarization of AAM. Several studies have reported that the activity of AAM can promote tissue-repair functions such as cell proliferation or matrix remodeling through the expression of different molecules such as arginase, IL-10, TGFβ1, Ym1, and Fizz1 (47, 48, 84). In our study, using an *in vitro* culture system, we demonstrated that CHR-treated AAM conditioned medium preserved gene expression of TJ proteins in LPS- and DSS-stimulated epithelial cells and improved the functional capacities of epithelial cells by regulating migration, proliferation, and viability. This is supported by previous data demonstrating that a reduction of intestinal inflammation is associated with an enhancement of AAM activity, limitation of the proinflammatory signals, and maintenance of IECs functions (85, 86). Furthermore, we demonstrated that CHR can directly restore the epithelial homeostasis by maintaining gene expression of TJ proteins and by improving the epithelial cells functional abilities to migrate, proliferate, and survive in response to LPS, DSS, or oxidative stress stimuli. Similar study demonstrated a protective effects of 5-hydroxytryptamine receptor 4 agonist against DSS-induced colitis, involving resistance of caco-2 epithelial cells to the detrimental effects of oxidative stress by the free radical donor (H_2_O_2_) (64).

Our study also described the ability of CHR to improve proliferation and viability of Caco-2 epithelial cells. Receptors for CHGA-derived peptides seem not to exist, but the sequence similarity of these peptides with cell penetrating abilities (70–72) may explain the ability of CHR to enter the cell and interact with the intracellular pathways. We speculate that CHR might affect some specific intracellular pathways including the p38 MAP kinase or the activator of transcription 1 (STAT1), which are well known to enhance the functional abilities of epithelial cells to proliferate and migrate (87, 88). Supporting this idea, recent studies have demonstrated the importance of these two pathways. Treatment of Caco-2 with pregnane X receptor agonists or IL-28 significantly increased wound healing activity and proliferation, and in both context, when give to mice, a significant decrease of colitis was determined (88, 89). Other pathways are also for consideration as CHR induces calcium entry in human neutrophils through a calmodulin-regulated calcium independent phospholipase A2 (70, 72), as this enzyme seems to play a role in the regulation of the integrity of epithelial TJ proteins and the pathogenesis of colitis (90).

Our findings also revealed that CHR is negatively correlated with IL-8 and IL-18 in colonic biopsies from patients with active UC. In parallel using our mouse model of colitis, we demonstrated that exogenous CHR treatment reduced the weight loss and colonic IL-18 release. Moreover, CHR peptide directly and indirectly through CHR-treated AAM conditioned medium decreased IL-8 and IL-18 release in LPS- and DSS-stimulated epithelial cell line. Studies have reported that a deletion of IL-18 protected against experimental colitis and minimized the mucosal damage through maintenance of the epithelium equilibrium (46, 91), demonstrating the importance of IL-18. The overall effect of CHR on IL-18 can also explain indirectly the impact of fibrosis described above, as transmural intestinal inflammation favors colitis-associated fibrosis through the promotion and the expression of collagen and IL-18 (92). Downregulation of IL-18 expression results in a decreased inflammatory process (92). IL-8 known as CXCL-8, is a potent chemoattractant secreted by IECs, and mediates polymorphonuclear leukocytes recruitment from the lamina propria to the epithelium and is increased during IBD (93, 94). The close relation between IL-8 and CHGA-derived peptides is supported by previous data demonstrating that vasostatin-1, another CHGA-derived peptide, can decrease the onset and severity of experimental colitis *via* an inhibition of human IECs IL-8 production (95). As in mice, the homolog of human *IL-8* is completely absent from their genome IL-8 was not quantified (93).

In this study, we assessed only the mRNA level considering the main concept of molecular biology, which states that “DNA makes RNA makes proteins,” suggesting a direct association between mRNA and protein levels (96). Although, several studies have found significant correlations between mRNA levels and protein levels (96–98), in some conditions the mRNA levels do not correlate with protein expression levels or even with the protein function. Therefore, the gene expression presented in our study can only provide an idea of understanding the potential action of CHR involved in the protection against colitis, and further studies are warranted to investigate the precise effects of CHR on the protein expression and localization of the proteins studied.

We cannot rule out the possibility that other mechanisms, including gut microbiota dysbiosis, apoptosis, and permeability, can also contribute to the changes seen post-treatment. Several studies have highlighted the importance of gut microbiota in IBD pathophysiology, innate immunity, and epithelial homeostasis (99–101). Previous studies have demonstrated that CHR features antimicrobial activity (25, 26) that may affect the gut microbiota and their associated metabolites. Consequently, future studies are required to investigate the potential effect of CHR on gut microbiota and also to confirm its role on intestinal permeability and apoptosis.

## Conclusion

**Figure 11:**
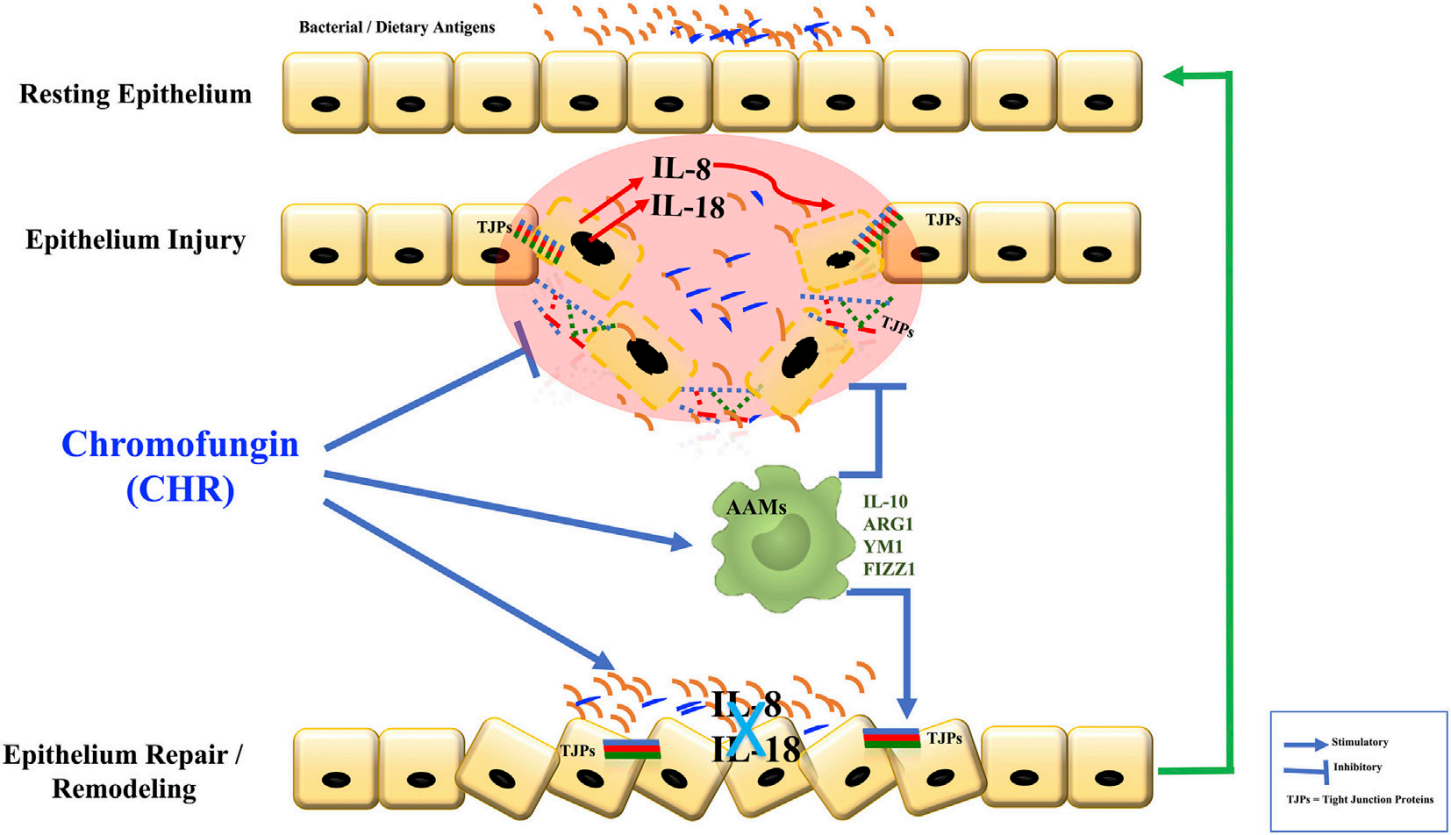
Graphical summary. Chromofungin (CHR) decreases tissue damage by the promotion of alternatively activated macrophages (AAM) macrophages that anti-inflammatory and regulatory molecules to decrease the onset of inflammation, reduces interleukin (IL)-8 and IL-18 release, maintains the tight junction (TJ) protein, and promotes the mucosal healing.

Here, we report a protective effect of CHR during the development of colonic inflammation (Figure 11). The results of this study have clinical relevance. First, they prompt close consideration of the relationship between CHR and disease activity in patients with IBD. Second, if this correlation is confirmed, then patients with IBD might be an appropriate group to target with novel treatment strategies involving CHR.

## Ethics Statement

Human subjects: patients diagnosed with active UC and persons with no IBD who were undergoing colonoscopy were recruited from the University of Manitoba IBD Clinical and Research Centre. Informed consent was obtained from patients and control subjects before the study. This study was approved by the University of Manitoba Health Research Ethics Board [HS14878 (E)]. Animals: experiments were approved by the University of Manitoba Animal Ethics Committee (Protocol # 15-010) and conducted under the Canadian guidelines for animal research.

## Author Contributions

Conceived and designed the experiments: NE and JEG. Collected the human tissue and data: CNB. Performed the experiments: NE. Analyzed and interpreted the data: NE. Revised the data analysis and interpretation: NE, CNB, and JEG. Performed research: HH, LK, JG, and MM. Contributed reagents/materials/analysis tools: JEG. Wrote the paper: NE and JEG. All authors have read and approved the manuscript.

## Conflict of Interest Statement

CNB has served on advisory boards or consulted to Abbvie Canada, Ferring Canada, Janssen Canada, Pfizer Canada, Shire Canada, Takeda Canada, Mylan Pharmaceuticals, and Napo Pharma and has received unrestricted educational grants from Abbvie Canada, Janssen Canada, Shire Canada, and Takeda Canada. The other authors declare that they have no conflicts of interest.

## References

[1] Xavier R Podolsky D. Unravelling the pathogenesis of inflammatory bowel disease. Nature (2007) 448(7152):427–34. 10.1038/nature06005 17653185

[2] Strober W Fuss I Mannon P. The fundamental basis of inflammatory bowel disease. J Clin Invest (2007) 117(3):514–21. 10.1172/JCI30587 17332878 PMC1804356

[3] Bouma G Strober W. The immunological and genetic basis of inflammatory bowel disease. Nat Rev Immunol (2003) 3(7):521–33. 10.1038/nri1132 12876555

[4] Molawi K Sieweke MH. Transcriptional control of macrophage identity, self-renewal, and function. Adv Immunol (2013) 120:269–300. 10.1016/B978-0-12-417028-5.00010-7 24070388

[5] Steinbach EC Plevy SE. The role of macrophages and dendritic cells in the initiation of inflammation in IBD. Inflamm Bowel Dis (2014) 20(1):166. 10.1097/MIB.0b013e3182a69dca 23974993 PMC4098861

[6] Gren ST Grip O. Role of monocytes and intestinal macrophages in Crohn’s disease and ulcerative colitis. Inflamm Bowel Dis (2016) 22(8):1992–8. 10.1097/MIB.0000000000000824 27243595

[7] Mills CD. Anatomy of a discovery: m1 and m2 macrophages. Front Immunol (2015) 6:212. 10.3389/fimmu.2015.00212 25999950 PMC4419847

[8] Mantovani A Biswas SK Galdiero MR Sica A Locati M. Macrophage plasticity and polarization in tissue repair and remodelling. J Pathol (2013) 229(2):176–85. 10.1002/path.4133 23096265

[9] Hunter MM Wang A Parhar KS Johnston MJ Van Rooijen N Beck PL In vitro-derived alternatively activated macrophages reduce colonic inflammation in mice. Gastroenterology (2010) 138(4):1395–405. 10.1053/j.gastro.2009.12.041 20044996

[10] Rizzo A Monteleone I Fina D Stolfi C Caruso R Fantini MC Inhibition of colitis by IL-25 associates with induction of alternatively activated macrophages. Inflamm Bowel Dis (2012) 18(3):449–59. 10.1002/ibd.21799 21688353

[11] Tu L Chen J Xu D Xie Z Yu B Tao Y IL-33-induced alternatively activated macrophage attenuates the development of TNBS-induced colitis. Oncotarget (2017) 8(17):27704. 10.18632/oncotarget.15984 28423665 PMC5438602

[12] Landy J Ronde E English N Clark SK Hart AL Knight SC Tight junctions in inflammatory bowel diseases and inflammatory bowel disease associated colorectal cancer. World J Gastroenterol (2016) 22(11):3117 10.3748/wjg.v22.i11.3117 27003989 PMC4789987

[13] Arnott I Kingstone K Ghosh S. Abnormal intestinal permeability predicts relapse in inactive Crohn disease. Scand J Gastroenterol (2000) 35(11):1163–9. 10.1080/003655200750056637 11145287

[14] Turner JR. Intestinal mucosal barrier function in health and disease. Nat Rev Immunol (2009) 9(11):799–809. 10.1038/nri2653 19855405

[15] Arai F Takahashi T Furukawa K Matsushima K Asakura H. Mucosal expression of interleukin-6 and interleukin-8 messenger RNA in ulcerative colitis and in Crohn’s disease. Dig Dis Sci (1998) 43(9):2071–9. 10.1023/A:1018815432504 9753275

[16] Struyf S Gouwy M Dillen C Proost P Opdenakker G Van Damme J. Chemokines synergize in the recruitment of circulating neutrophils into inflamed tissue. Eur J Immunol (2005) 35(5):1583–91. 10.1002/eji.200425753 15827963

[17] Shi X-Z Winston JH Sarna SK Differential immune and genetic responses in rat models of Crohn’s colitis and ulcerative colitis. Am J Physiol Gastrointest Liver Physiol (2011) 300(1):G41–51. 10.1152/ajpgi.00358.2010 20947704 PMC3025515

[18] D’amico MA Ghinassi B Izzicupo P Manzoli L Di Baldassarre A. Biological function and clinical relevance of chromogranin A and derived peptides. Endocr Connect (2014) 3(2):R45–54. 10.1530/EC-14-0027 24671122 PMC5395093

[19] Chanat E Huttner WB. Milieu-induced, selective aggregation of regulated secretory proteins in the trans-Golgi network. J Cell Biol (1991) 115(6):1505–19. 10.1083/jcb.115.6.1505 1757460 PMC2289203

[20] Loh YP Cheng Y Mahata SK Corti A Tota B. Chromogranin A and derived peptides in health and disease. J Mol Neurosci (2012) 48(2):347–56. 10.1007/s12031-012-9728-2 22388654 PMC3402615

[21] Metz-Boutigue MH Garcia-Sablone P Hogue-Angeletti R Aunis D. Intracellular and extracellular processing of chromogranin A. Determination of cleavage sites. Eur J Biochem (1993) 217(1):247–57. 10.1111/j.1432-1033.1993.tb18240.x 8223562

[22] Maget-Dana R. The monolayer technique: a potent tool for studying the interfacial properties of antimicrobial and membrane-lytic peptides and their interactions with lipid membranes. Biochim Biophys Acta (1999) 1462(1):109–40. 10.1016/S0005-2736(99)00203-5 10590305

[23] Yoo SH Identification of the calcium-dependent calmodulin-binding region of chromogranin A. Biochemistry (1992) 31(26):6134–40. 10.1021/bi00141a025 1627556

[24] Lugardon K Chasserot-Golaz S Kieffer A-E Maget-Dana R Nullans G Kieffer B Structural and biological characterization of chromofungin, the antifungal chromogranin A-(47–66)-derived peptide. J Biol Chem (2001) 276(38):35875–82. 10.1074/jbc.M104670200 11451958

[25] Metz-Boutigue M Zhang D Lavaux T Schneider F Aunis D Two chromogranin A-derived peptides, chromofungin and catestatin, induce neutrophil activation via a store-operated channel-dependent mechanism. Crit Care (2010) 14(Suppl 2):32 10.1186/cc9135

[26] Lugardon K Chasserot-Golaz S Kieffer A Maget-Dana R Nullans G Kieffer B Structural and biological characterization of chromofungin, the antifungal chromogranin A (47-66)-derived peptide. Ann N Y Acad Sci (2002) 971(1):359–61. 10.1111/j.1749-6632.2002.tb04496.x 12438152

[27] Ghia J-E Crenner F Metz-Boutigue M-H Aunis D Angel F Effects of a chromogranin-derived peptide (CgA 47–66) in the writhing nociceptive response induced by acetic acid in rats. Regul Pept (2004) 119(3):199–207. 10.1016/j.regpep.2004.02.014 15120481

[28] Filice E Pasqua T Quintieri AM Cantafio P Scavello F Amodio N Chromofungin, CgA47-66-derived peptide, produces basal cardiac effects and postconditioning cardioprotective action during ischemia/reperfusion injury. Peptides (2015) 71:40–8. 10.1016/j.peptides.2015.06.013 26151429

[29] Sidhu R Drew K McAlindon ME Lobo AJ Sanders DS Elevated serum chromogranin A in irritable bowel syndrome (IBS) and inflammatory bowel disease (IBD): a shared model for pathogenesis? Inflamm Bowel Dis (2010) 16(3):361 10.1002/ibd.20982 19575362

[30] Sciola V Massironi S Conte D Caprioli F Ferrero S Ciafardini C Plasma chromogranin A in patients with inflammatory bowel disease. Inflamm Bowel Dis (2009) 15(6):867–71. 10.1002/ibd.20851 19090560

[31] Zissimopoulos A Vradelis S Konialis M Chadolias D Bampali A Constantinidis T Chromogranin A as a biomarker of disease activity and biologic therapy in inflammatory bowel disease: a prospective observational study. Scand J Gastroenterol (2014) 49(8):942–9. 10.3109/00365521.2014.920910 24897131

[32] Rabbi MF Labis B Metz-Boutigue M-H Bernstein CN Ghia J-E. Catestatin decreases macrophage function in two mouse models of experimental colitis. Biochem Pharmacol (2014) 89(3):386–98. 10.1016/j.bcp.2014.03.003 24637240

[33] Eissa N Rabbi MF Munyaka PM Khafipour A Bernstein CN Ghia J-E Mo1929 critical role of chromogranin-A on macrophage intrinsic apoptotic pathway in colitis: human and animal studies. Gastroenterology (2016) 150(4):S819 10.1016/S0016-5085(16)32771-8

[34] Etienne O Gasnier C Taddei C Voegel J-C Aunis D Schaaf P Antifungal coating by biofunctionalized polyelectrolyte multilayered films. Biomaterials (2005) 26(33):6704–12. 10.1016/j.biomaterials.2005.04.068 15992921

[35] Metz-Boutigue MH Goumon Y Strub J Lugardon K Aunis D Antimicrobial chromogranins and proenkephalin-A-derived peptides. Ann N Y Acad Sci (2003) 992(1):168–78. 10.1111/j.1749-6632.2003.tb03147.x 12794056

[36] Okayasu I Hatakeyama S Yamada M Ohkusa T Inagaki Y Nakaya R A novel method in the induction of reliable experimental acute and chronic ulcerative colitis in mice. Gastroenterology (1990) 98(3):694–702. 10.1016/0016-5085(90)90290-H 1688816

[37] Cooper HS Murthy S Shah R Sedergran D. Clinicopathologic study of dextran sulfate sodium experimental murine colitis. Lab Invest (1993) 69(2):238–49. 8350599

[38] Johnson LA Luke A Sauder K Moons DS Horowitz JC Higgins PD Intestinal fibrosis is reduced by early elimination of inflammation in a mouse model of IBD: impact of a “Top-Down” approach to intestinal fibrosis in mice. Inflamm Bowel Dis (2012) 18(3):460–71. 10.1002/ibd.21812 21761511 PMC3206985

[39] Ding S Walton KL Blue RE MacNaughton K Magness ST Lund PK. Mucosal healing and fibrosis after acute or chronic inflammation in wild type FVB-N mice and C57BL6 procollagen α1 (I)-promoter-GFP reporter mice. PLoS One (2012) 7(8):e42568. 10.1371/journal.pone.0042568 22880035 PMC3411826

[40] Mosser DM Zhang X Activation of murine macrophages. Current Protocols in Immunology. (Chap. 14), (2008). Unit 14.2. 10.1002/0471142735.im1402s83 PMC2822273 19016446

[41] Walsh-Reitz MM Huang EF Musch MW Chang EB Martin TE Kartha S AMP-18 protects barrier function of colonic epithelial cells: role of tight junction proteins. Am J Physiol Gastrointest Liver Physiol (2005) 289(1):G163–71. 10.1152/ajpgi.00013.2005 15961882 PMC1444946

[42] Schneider CA Rasband WS Eliceiri KW NIH Image to ImageJ: 25 years of image analysis. Nat Methods (2012) 9(7):671 10.1038/nmeth.2089 22930834 PMC5554542

[43] Eissa N Hussein H Wang H Rabbi MF Bernstein CN Ghia J-E. Stability of reference genes for messenger RNA quantification by real-time PCR in mouse dextran sodium sulfate experimental colitis. PLoS One (2016) 11(5):e0156289. 10.1371/journal.pone.0156289 27244258 PMC4886971

[44] Eissa N Kermarrec L Hussein H Bernstein CN Ghia J-E. Appropriateness of reference genes for normalizing messenger RNA in mouse 2, 4-dinitrobenzene sulfonic acid (DNBS)-induced colitis using quantitative real time PCR. Sci Rep (2017) 7:42427. 10.1038/srep42427 28186172 PMC5301225

[45] Eissa N Hussein H Rabbi MF Munyaka PM Khafipour A Bernstein CN Tu1832 stability of reference genes for messenger RNA quantification by real-time PCR in mouse dextran sodium sulfate experimental colitis. Gastroenterology (2016) 150(4):S955–6. 10.1016/S0016-5085(16)33226-7 PMC4886971 27244258

[46] Sivakumar P Westrich G Kanaly S Garka K Born T Derry J Interleukin 18 is a primary mediator of the inflammation associated with dextran sulphate sodium induced colitis: blocking interleukin 18 attenuates intestinal damage. Gut (2002) 50(6):812–20. 10.1136/gut.50.6.812 12010883 PMC1773244

[47] Mosser DM Edwards JP. Exploring the full spectrum of macrophage activation. Nat Rev Immunol (2008) 8(12):958–69. 10.1038/nri2448 19029990 PMC2724991

[48] Novak ML Koh TJ Macrophage phenotypes during tissue repair. J Leukoc Biol (2013) 93(6):875–81. 10.1189/jlb.1012512 23505314 PMC3656331

[49] Eissa N Ghia J. Immunomodulatory effect of ghrelin in the intestinal mucosa. Neurogastroenterol Motil (2015) 27(11):1519–27. 10.1111/nmo.12703 26503163

[50] Eissa N Kermarrec L Metz-Boutigue M-H Hendy GN Bernstein CN Ghia J-E 654-Chromofungin treatment promotes alternatively activated macrophages, suppresses classically activated macrophages and improves epithelial cell functions during colitis. Gastroenterology (2017) 152(5):S143 10.1016/S0016-5085(17)30806-5

[51] Rabbi MF Eissa N Munyaka PM Khafipour A Khafipour E Ghia J-E Tu1893 human catestatin represses reactivation of intestinal inflammation in a murine model of colitis through the M1 macrophages and not the gut microbiota. Gastroenterology (2016) 150(4):S969 10.1016/S0016-5085(16)33286-3

[52] Eissa N Rabbi M Bernstein C Ghia J Chromofungin & pancreastatin co-regulate migration and functional plasticity of murine peritoneal macrophages. Neurogastroenterol Motil (2016) 28:103–4. 10.1111/nmo.12881

[53] Tanoue T Nishitani Y Kanazawa K Hashimoto T Mizuno M. In vitro model to estimate gut inflammation using co-cultured Caco-2 and RAW264. 7 cells. Biochem Biophys Res Commun (2008) 374(3):565–9. 10.1016/j.bbrc.2008.07.063 18647595

[54] Araki Y Sugihara H Hattori T. In vitro effects of dextran sulfate sodium on a Caco-2 cell line and plausible mechanisms for dextran sulfate sodium-induced colitis. Oncol Rep (2006) 16(6):1357–62. 10.3892/or.16.6.1357 17089061

[55] Hollebeeck S Winand J Hérent M-F During A Leclercq J Larondelle Y Anti-inflammatory effects of pomegranate (Punica granatum L.) husk ellagitannins in Caco-2 cells, an in vitro model of human intestine. Food Funct (2012) 3(8):875–85. 10.1039/c2fo10258g 22733173

[56] Leonard F Ali H Collnot E-M Crielaard BJ Lammers T Storm G Screening of budesonide nanoformulations for treatment of inflammatory bowel disease in an inflamed 3D cell-culture model. ALTEX (2012) 29(3):275. 10.14573/altex.2012.3.275 22847255

[57] Le Ferrec E Chesne C Artusson P Brayden D Fabre G Gires P In vitro models of the intestinal barrier. The report and recommendations of ECVAM Workshop 46. European Centre for the Validation of Alternative Methods. Altern Lab Anim (2001) 29:649–68. 11709041 10.1177/026119290102900604

[58] Deferme S Annaert P Augustijns P In vitro screening models to assess intestinal drug absorption and metabolism. In: Ehrhardt C Kim KJ editors. Drug Absorption Studies. Biotechnology: Pharmaceutical Aspects. Vol VII Boston, MA: Springer (2008). p. 182–215.

[59] Shah P Jogani V Bagchi T Misra A. Role of Caco-2 cell monolayers in prediction of intestinal drug absorption. Biotechnol Prog (2006) 22(1):186–98. 10.1021/bp050208u 16454510

[60] Leonard F Collnot E-M Lehr C-M. A three-dimensional coculture of enterocytes, monocytes and dendritic cells to model inflamed intestinal mucosa in vitro. Mol Pharm (2010) 7(6):2103–19. 10.1021/mp1000795 20809575

[61] Bain CC Mowat AM. Macrophages in intestinal homeostasis and inflammation. Immunol Rev (2014) 260(1):102–17. 10.1111/imr.12192 24942685 PMC4141699

[62] Haribhai D Ziegelbauer J Jia S Upchurch K Yan K Schmitt EG Alternatively activated macrophages boost induced regulatory T and Th17 cell responses during immunotherapy for colitis. J Immunol (2016) 196(8):3305–17. 10.4049/jimmunol.1501956 26927797 PMC4851766

[63] Lissner D Schumann M Batra A Kredel L-I Kühl AA Erben U Monocyte and M1 macrophage-induced barrier defect contributes to chronic intestinal inflammation in IBD. Inflamm Bowel Dis (2015) 21(6):1297–305. 10.1097/MIB.0000000000000384 25901973 PMC4450953

[64] Spohn SN Bianco F Scott RB Keenan CM Linton AA O’Neill CH Protective actions of epithelial 5-hydroxytryptamine 4 receptors in normal and inflamed colon. Gastroenterology (2016) 151(5):933–44.e3. 10.1053/j.gastro.2016.07.032 27480173 PMC5159265

[65] Zhang D The Expression and Role of Chromogranin A and Its Derived Peptides in Septic Patients [Doctoral Dissertation]. Strasbourg: Université Louis Pasteur (2008).

[66] Bauer C Duewell P Mayer C Lehr HA Fitzgerald KA Dauer M Colitis induced in mice with dextran sulfate sodium (DSS) is mediated by the NLRP3 inflammasome. Gut (2010) 59(9):1192–9. 10.1136/gut.2009.197822 20442201

[67] Wang W Li X Zheng D Zhang D Huang S Zhang X Dynamic changes of peritoneal macrophages and subpopulations during ulcerative colitis to metastasis of colorectal carcinoma in a mouse model. Inflamm Res (2013) 62(7):669–80. 10.1007/s00011-013-0619-y 23625042

[68] Gonzalez-Rey E Anderson P Gonzalez MA Rico L Buscher D Delgado M. Human adult stem cells derived from adipose tissue protect against experimental colitis and sepsis. Gut (2009) 58:929–39. 10.1136/gut.2008.168534 19136511

[69] Rabbi M Eissa N Munyaka P Kermarrec L Elgazzar O Khafipour E Reactivation of intestinal inflammation is suppressed by catestatin in a murine model of colitis via M1 macrophages and not the gut microbiota. Front Immunol (2017) 8:985 10.3389/fimmu.2017.00985 28871257 PMC5566981

[70] Zhao E Zhang D Basak A Trudeau VL. New insights into granin-derived peptides: evolution and endocrine roles. Gen Comp Endocrinol (2009) 164(2):161–74. 10.1016/j.ygcen.2009.01.011 19523383

[71] Henriques ST Melo MN Castanho MA. Cell-penetrating peptides and antimicrobial peptides: how different are they? Biochem J (2006) 399(1):1–7. 10.1042/BJ20061100 16956326 PMC1570158

[72] Zhang D Shooshtarizadeh P Laventie B-J Colin DA Chich J-F Vidic J Two chromogranin A-derived peptides induce calcium entry in human neutrophils by calmodulin-regulated calcium independent phospholipase A2. PLoS One (2009) 4(2):e4501. 10.1371/journal.pone.0004501 19225567 PMC2639705

[73] Neurath MF. Cytokines in inflammatory bowel disease. Nat Rev Immunol (2014) 14(5):329–42. 10.1038/nri3661 24751956

[74] Zhu Y Li X Chen J Chen T Shi Z Lei M The pentacyclic triterpene Lupeol switches M1 macrophages to M2 and ameliorates experimental inflammatory bowel disease. Int Immunopharmacol (2016) 30:74–84. 10.1016/j.intimp.2015.11.031 26655877

[75] Prasse A Pechkovsky DV Toews GB Jungraithmayr W Kollert F Goldmann T A vicious circle of alveolar macrophages and fibroblasts perpetuates pulmonary fibrosis via CCL18. Am J Respir Crit Care Med (2006) 173(7):781–92. 10.1164/rccm.200509-1518OC 16415274

[76] Fairweather D Cihakova D. Alternatively activated macrophages in infection and autoimmunity. J Autoimmun (2009) 33(3):222–30. 10.1016/j.jaut.2009.09.012 19819674 PMC2783278

[77] Anthony RM Urban JF Jr Alem F Hamed HA Rozo CT Boucher J-L Memory TH2 cells induce alternatively activated macrophages to mediate protection against nematode parasites. Nat Med (2006) 12(8):955 10.1038/nm1451 16892038 PMC1955764

[78] Schnoor M Cullen P Lorkowski J Stolle K Robenek H Troyer D Production of type VI collagen by human macrophages: a new dimension in macrophage functional heterogeneity. J Immunol (2008) 180(8):5707–19. 10.4049/jimmunol.180.8.5707 18390756

[79] Yang M Zheng J Miao Y Wang Y Cui W Guo J Serum-glucocorticoid regulated kinase 1 regulates alternatively activated macrophage polarization contributing to angiotensin II-induced inflammation and cardiac fibrosis. Arterioscler Thromb Vasc Biol (2012) 32(7):1675–86. 10.1161/ATVBAHA.112.248732 22556335

[80] Pesce JT Ramalingam TR Mentink-Kane MM Wilson MS El Kasmi KC Smith AM Arginase-1-expressing macrophages suppress Th2 cytokine-driven inflammation and fibrosis. PLoS Pathog (2009) 5(4):e1000371. 10.1371/journal.ppat.1000371 19360123 PMC2660425

[81] Thomas JA Pope C Wojtacha D Robson AJ Gordon-Walker TT Hartland S Macrophage therapy for murine liver fibrosis recruits host effector cells improving fibrosis, regeneration, and function. Hepatology (2011) 53(6):2003–15. 10.1002/hep.24315 21433043

[82] Leung G Wang A Fernando M Phan VC McKay DM. Bone marrow-derived alternatively activated macrophages reduce colitis without promoting fibrosis: participation of IL-10. Am J Physiol Gastrointest Liver Physiol (2013) 304(9):G781–92. 10.1152/ajpgi.00055.2013 23494123

[83] Stremmel W Staffer S Schneider MJ Gan-Schreier H Wannhoff A Stuhrmann N Genetic mouse models with intestinal-specific tight junction deletion resemble an ulcerative colitis phenotype. J Crohns Colitis (2017). 10.1093/ecco-jcc/jjx075 28575164 PMC5881657

[84] Rőszer T. Understanding the mysterious M2 macrophage through activation markers and effector mechanisms. Mediators Inflamm (2015) 2015:816460. 10.1155/2015/816460 26089604 PMC4452191

[85] Jang S-E Han MJ Kim S-Y Kim D-H. *Lactobacillus plantarum* CLP-0611 ameliorates colitis in mice by polarizing M1 to M2-like macrophages. Int Immunopharmacol (2014) 21(1):186–92. 10.1016/j.intimp.2014.04.021 24815859

[86] Kawano M Miyoshi M Ogawa A Sakai F Kadooka Y. *Lactobacillus gasseri* SBT2055 inhibits adipose tissue inflammation and intestinal permeability in mice fed a high-fat diet. J Nutr Sci (2016) 5:e23. 10.1017/jns.2016.12 27293560 PMC4891558

[87] Chaturvedi LS Marsh HM Basson MD. Role of RhoA and its effectors ROCK and mDia1 in the modulation of deformation-induced FAK, ERK, p38, and MLC motogenic signals in human Caco-2 intestinal epithelial cells. Am J Physiol Cell Physiol (2011) 301(5):C1224–38. 10.1152/ajpcell.00518.2010 21849669 PMC3213924

[88] Chiriac MT Buchen B Wandersee A Hundorfean G Günther C Bourjau Y Activation of epithelial signal transducer and activator of transcription 1 by interleukin 28 controls mucosal healing in mice with colitis and is increased in mucosa of patients with inflammatory bowel disease. Gastroenterology (2017) 153(1):123–38. 10.1053/j.gastro.2017.03.015 28342759

[89] Terc J Hansen A Alston L Hirota SA Pregnane X receptor agonists enhance intestinal epithelial wound healing and repair of the intestinal barrier following the induction of experimental colitis. Eur J Pharmaceut Sci (2014) 55:12–9. 10.1016/j.ejps.2014.01.007 24486481

[90] Chelakkot C Ghim J Rajasekaran N Choi J-S Kim J-H Jang MH Intestinal epithelial cell-specific deletion of PLD2 alleviates DSS-induced colitis by regulating occludin. Sci Rep (2017) 7(1):1573. 10.1038/s41598-017-01797-y 28484281 PMC5431506

[91] Nowarski R Jackson R Gagliani N De Zoete MR Palm NW Bailis W Epithelial IL-18 equilibrium controls barrier function in colitis. Cell (2015) 163(6):1444–56. 10.1016/j.cell.2015.10.072 26638073 PMC4943028

[92] Peterson TC Peterson MR Raoul JM. The effect of pentoxifylline and its metabolite-1 on inflammation and fibrosis in the TNBS model of colitis. Eur J Pharmacol (2011) 662(1):47–54. 10.1016/j.ejphar.2011.04.030 21554874

[93] Asfaha S Dubeykovskiy AN Tomita H Yang X Stokes S Shibata W Mice that express human interleukin-8 have increased mobilization of immature myeloid cells, which exacerbates inflammation and accelerates colon carcinogenesis. Gastroenterology (2013) 144(1):155–66. 10.1053/j.gastro.2012.09.057 23041326 PMC3990262

[94] Ina K Kusugami K Yamaguchi T Imada A Hosokawa T Ohsuga M Mucosal interleukin-8 is involved in neutrophil migration and binding to extracellular matrix in inflammatory bowel disease. Am J Gastroenterol (1997) 92(8):1342–6. 9260803

[95] Rumio C Dusio G Colombo B Gasparri A Cardani D Marcucci F The N-terminal fragment of chromogranin A, vasostatin-1 protects mice from acute or chronic colitis upon oral administration. Dig Dis Sci (2012) 57(5):1227–37. 10.1007/s10620-012-2031-9 22278339

[96] Gry M Rimini R Strömberg S Asplund A Pontén F Uhlén M Correlations between RNA and protein expression profiles in 23 human cell lines. BMC Genomics (2009) 10(1):365. 10.1186/1471-2164-10-365 19660143 PMC2728742

[97] Fu N Drinnenberg I Kelso J Wu J-R Pääbo S Zeng R Comparison of protein and mRNA expression evolution in humans and chimpanzees. PLoS One (2007) 2(2):e216. 10.1371/journal.pone.0000216 17299596 PMC1789144

[98] Greenbaum D Colangelo C Williams K Gerstein M. Comparing protein abundance and mRNA expression levels on a genomic scale. Genome Biol (2003) 4(9):117. 10.1186/gb-2003-4-9-117 12952525 PMC193646

[99] Munyaka PM Eissa N Bernstein CN Khafipour E Ghia J-E. Antepartum antibiotic treatment increases offspring susceptibility to experimental colitis: a role of the gut microbiota. PLoS One (2015) 10(11):e0142536. 10.1371/journal.pone.0142536 26605545 PMC4659638

[100] Munyaka PM Khafipour A Wang H Eissa N Khafipour E Ghia J-E Mo1774 prenatal antibiotic treatment increases offspring’s susceptibility to experimental colitis: a role of the gut microbiota. Gastroenterology (2015) 148(4):S–708. 10.1016/S0016-5085(15)32404-5 PMC4659638 26605545

[101] Rabbi MF Munyaka PM Eissa N Metz-Boutigue M-H Khafipour E Ghia JE Human catestatin alters gut microbiota composition in mice. Front Microbiol (2016) 7:2151 10.3389/fmicb.2016.02151 28144234 PMC5239785

